# Urine NGAL as a biomarker for septic AKI: a critical appraisal of clinical utility—data from the observational FINNAKI study

**DOI:** 10.1186/s13613-020-00667-7

**Published:** 2020-04-28

**Authors:** Sanna Törnblom, Sara Nisula, Liisa Petäjä, Suvi T. Vaara, Mikko Haapio, Eero Pesonen, Ville Pettilä, the FINNAKI study group, Raili Laru-Sompa, Anni Pulkkinen, Minna Saarelainen, Mikko Reilama, Sinikka Tolmunen, Ulla Rantalainen, Marja Miettinen, Markku Suvela, Katrine Pesola, Pekka Saastamoinen, Sirpa Kauppinen, Ville Pettilä, Kirsi-Maija Kaukonen, Anna-Maija Korhonen, Sara Nisula, Suvi Vaara, Raili Suojaranta-Ylinen, Leena Mildh, Mikko Haapio, Laura Nurminen, Sari Sutinen, Leena Pettilä, Helinä Laitinen, Heidi Syrjä, Kirsi Henttonen, Elina Lappi, Hillevi Boman, Tero Varpula, Päivi Porkka, Mirka Sivula, Mira Rahkonen, Anne Tsurkka, Taina Nieminen, Niina Prittinen, Ari Alaspää, Ville Salanto, Hanna Juntunen, Teija Sanisalo, Ilkka Parviainen, Ari Uusaro, Esko Ruokonen, Stepani Bendel, Niina Rissanen, Maarit Lång, Sari Rahikainen, Saija Rissanen, Merja Ahonen, Elina Halonen, Eija Vaskelainen, Meri Poukkanen, Esa Lintula, Sirpa Suominen, Jorma Heikkinen, Timo Lavander, Kirsi Heinonen, Anne-Mari Juopperi, Tadeusz Kaminski, Fiia Gäddnäs, Tuija Kuusela, Jane Roiko, Sari Karlsson, Matti Reinikainen, Tero Surakka, Helena Jyrkönen, Tanja Eiserbeck, Jaana Kallinen, Vesa Lund, Päivi Tuominen, Pauliina Perkola, Riikka Tuominen, Marika Hietaranta, Satu Johansson, Seppo Hovilehto, Anne Kirsi, Pekka Tiainen, Tuija Myllärinen, Pirjo Leino, Anne Toropainen, Anne Kuitunen, Ilona Leppänen, Markus Levoranta, Sanna Hoppu, Jukka Sauranen, Jyrki Tenhunen, Atte Kukkurainen, Samuli Kortelainen, Simo Varila, Outi Inkinen, Niina Koivuviita, Jutta Kotamäki, Anu Laine, Tero Ala-Kokko, Jouko Laurila, Sinikka Sälkiö, Simo-Pekka Koivisto, Raku Hautamäki, Maria Skinnar

**Affiliations:** 1 Division of Intensive Care Medicine, Department of Anaesthesiology, Intensive Care and Pain Medicine, University of Helsinki and Helsinki University Hospital, PO Box 340, 00029 HUS Helsinki, Finland; 2 Division of Anaesthesiology, Department of Anaesthesiology, Intensive Care and Pain Medicine, University of Helsinki and Helsinki University Hospital, Helsinki, Finland; 3 Nephrology, University of Helsinki and Helsinki University Hospital, Helsinki, Finland

**Keywords:** Neutrophil gelatinase-associated lipocalin, Acute kidney injury, Sepsis, Critical illness, Intensive care

## Abstract

**Background:**

Neutrophil gelatinase-associated lipocalin (NGAL) is released from kidney tubular cells under stress as well as from neutrophils during inflammation. It has been suggested as a biomarker for acute kidney injury (AKI) in critically ill patients with sepsis. To evaluate clinical usefulness of urine NGAL (uNGAL), we post-hoc applied recently introduced statistical methods to a sub-cohort of septic patients from the prospective observational Finnish Acute Kidney Injury (FINNAKI) study. Accordingly, in 484 adult intensive care unit patients with sepsis by Sepsis-3 criteria, we calculated areas under the receiver operating characteristic curves (AUCs) for the first available uNGAL to assess discrimination for four outcomes: AKI defined by Kidney Disease: Improving Global Outcomes (KDIGO) criteria, severe (KDIGO 2–3) AKI, and renal replacement therapy (RRT) during the first 3 days of intensive care, and mortality at day 90. We constructed clinical prediction models for the outcomes and used risk assessment plots and decision curve analysis with predefined threshold probabilities to test whether adding uNGAL to the models improved reclassification or decision making in clinical practice.

**Results:**

Incidences of AKI, severe AKI, RRT, and mortality were 44.8% (217/484), 27.7% (134/484), 9.5% (46/484), and 28.1% (136/484). Corresponding AUCs for uNGAL were 0.690, 0.728, 0.769, and 0.600. Adding uNGAL to the clinical prediction models improved discrimination of AKI, severe AKI, and RRT. However, the net benefits for the new models were only 1.4% (severe AKI and RRT) to 2.5% (AKI), and the number of patients needed to be tested per one extra true-positive varied from 40 (AKI) to 74 (RRT) at the predefined threshold probabilities.

**Conclusions:**

The results of the recommended new statistical methods do not support the use of uNGAL in critically ill septic patients to predict AKI or clinical outcomes.

## Background

Neutrophil gelatinase-associated lipocalin (NGAL) has been studied extensively as a biomarker for detection and evolution of acute kidney injury (AKI) as well as outcome [1, 2]. NGAL is a protein first found in neutrophil granules [3], but synthesized in numerous human tissues in addition to kidney epithelium—e.g., respiratory tract, stomach, and colon. All in vivo functions of NGAL are not plausibly unraveled. It increases rapidly in serum and urine not only in conjunction with renal tubular injury, but also in bacterial infections, non-infectious systemic inflammatory response syndrome, and chronic and systemic diseases without bacterial infection [4]. Consequently, inflammation is considered a confounding factor hindering the routine use of NGAL as a biomarker of AKI in intensive care patients with sepsis [5–7].

In a recent meta-analysis, urine NGAL (uNGAL) predicted septic AKI with an area under the receiver operating characteristic curve (AUC) of 0.90 [8], but the individual studies were rather small, the sample sizes varying between 45 and 168. Besides, generalizability of the meta-analysis may be questioned since 65% of the sepsis patients were from Asia. Furthermore, currently used statistical methods have several shortcomings: AUCs are not very suitable for evaluating the incremental value of biomarkers [9] or assessing clinical usefulness [10]. Newer reclassification methods may even make useless biomarkers appear applicable [11]. Although there is obvious need for better tools than urine output and serum creatinine for early detection and classification of AKI, the existing data on any kidney injury biomarker for AKI diagnosis, staging, prognosis, or treatment are inadequate [12].

We have previously tested the ability of uNGAL to predict AKI, renal replacement therapy (RRT), and 90-day mortality in a large non-selected cohort of 1042 adult intensive care patients [13]. Patients with sepsis comprised 46% of the study population. In comparison to the previous meta-analysis [8], this is by far the largest cohort of septic patients with uNGAL measurements. Since we did not report the septic patients separately, they could not be included in the meta-analysis [8]. We now extended our analyses to evaluate the usefulness of uNGAL in predicting AKI, RRT, and 90-day mortality in septic patients using more sophisticated statistical methods: risk assessment plot (RAP) [14] and decision curve analysis (DCA) [10]. Accordingly, we tested the hypothesis that uNGAL improves the performance of clinical risk models for AKI, RRT, and 90-day mortality in a homogeneous and clinically important group of critically ill septic patients using these new statistical methods. We are not aware of a similar detailed analysis of uNGAL or its clinical usefulness in the literature.

## Methods

### Patients

We analyzed the urine of septic patients of this FINNAKI NGAL—substudy [13]. The Ethics Committee of the Department of Surgery in Helsinki University Hospital gave a nationwide approval for the FINNAKI study [15] with a deferred consent policy, confirmed by a written consent from each patient or his/her proxy.

### Data

The patients of the original study [13] were prospectively screened for sepsis defined by the American College of Chest Physicians/Society of Critical Care Medicine (ACCP/SCCM) criteria [16]. To increase the generalizability of the results, we now reclassified these patients using the recent Third International Consensus Definitions for Sepsis and Septic Shock (Sepsis-3) criteria [17]. We included patients fulfilling the criteria during the first 3 days of intensive care.

We defined AKI according to Kidney Disease: Improving Global Outcomes (KDIGO) criteria including both plasma creatinine and urine output criteria [18]. Urine output was measured hourly and plasma creatinine daily. The last available plasma creatinine value from the preceding year up to 1 week before intensive care unit (ICU) admission was used as the baseline value. When not available, we estimated the baseline creatinine value using the Modification of Diet in Renal Disease equation assuming a glomerular filtration rate of 75 ml/min/1.73 m^2^ [19]. We included data on AKI and RRT during the first 3 days of ICU stay, choosing the highest stage for the final KDIGO stage of each patient for the analyses. We obtained 90-day mortality data from the Finnish Population Register Centre.

### Measurement of uNGAL

The urine samples were collected on ICU admission (0 h), at 12 and 24 h, and stored as described elsewhere [13]. The person who analyzed the samples in duplicate with NGAL ELISA Rapid Kit (BioPorto^®^ Gentofte, Denmark) was blinded to patient records. The validated enzyme-linked immunosorbent assay (ELISA) method has a good intra- and inter-assay precision [20] and a measurement range of 10–1000 ng/ml. For the analyses, values below 10 ng/ml were registered as the lowest measurable value (10 ng/ml) and values above 1000 ng/ml as the highest measurable value (1000 ng/ml). For predictive calculations, we chose the first available uNGAL measurement (0, 12 or 24 h) for each patient.

### Statistical analyses

We tested four different outcomes: (1) AKI by original KDIGO classification (KDIGO stages 1–3), (2) “severe” AKI (KDIGO stages 2–3), (3) RRT, and (4) 90-day mortality. To simulate clinical decision making, we constructed clinical risk models for these outcomes using clinical variables available at the time of ICU admission. We tested associations of these variables with the outcomes using Mann–Whitney *U*, Chi square or Fisher’s exact test (with a two-sided *p* value), as appropriate. We conducted multivariable logistic regression analyses entering variables with the strongest associations (shown in Additional file 1: Table S1) simultaneously. We restricted the number of covariates to 1 per 8 dependent endpoints to avoid overfitting [21] and imputed missing values (Additional file 1: Table S1) as recommended [22]. To ensure that the assumptions for multivariable logistic regression were met, we checked the correlations between the variables and conducted multiple regression analysis to rule out multicollinearity (Additional file 2). We used Hosmer–Lemeshow test to evaluate model goodness of fit. Thereafter, we added uNGAL to the clinical risk models and gained new risk models for the four outcomes (Additional file 3). We calculated AUCs with 95% confidence intervals (CIs) for uNGAL alone, for the clinical risk models, and for the new risk models including uNGAL. To evaluate the predictive value of uNGAL, we calculated category-free net reclassification improvement (cfNRI) [23] and integrated discrimination improvement (IDI) [24], and draw RAPs [14] for each outcome. We describe these metrics in detail in Additional file 4: Statistical methods.

We also conducted DCAs [10] for the outcomes to illustrate the net benefit of adding uNGAL to the clinical prediction models. DCA plots net benefit against threshold probability. Net benefit delineates gained new true-positive results without false-positive results and varies according to the chosen threshold probability, that is, the probability above which the patient is offered treatment (e.g., ICU admission). For example, with threshold probabilities of 0, 1, or 0.1, we would admit all patients, none of the patients, or those having a risk of ≥ 10%, respectively. A threshold probability is chosen according to the significance of false-negative versus false-positive results. Threshold probability of 0.1 means that we consider the harm of a false-negative result (denial of necessary ICU admission) 9 times (1–0.1/0.1) worse than a false-positive result (unnecessary ICU admission). For more serious outcomes, false-negative results are considered more harmful and the threshold lowered. Accordingly, for AKI, severe AKI, RRT, and 90-day mortality, we prospectively chose threshold probabilities of 0.3, 0.2, 0.1, and 0.05, respectively. We calculated test trade-offs to determine the minimum number of patients to be tested per one extra true-positive classification [25]. Finally, we performed a sensitivity analysis excluding patients that did not have 0-h urine sample.

We present the data as medians with interquartile ranges (IQRs) or absolute numbers (percentage with 95% CIs). Statistical analyses were conducted using SPSS 22 software (SPSS Inc., Chicago, IL, USA), MedCalc Statistical Software version 18 (MedCalc Software bvba, Ostend, Belgium; http://www.medcalc.org; 2018) and R 3.4.3 (R Development Core Team, Vienna, Austria).

## Results

**Fig. 1:**
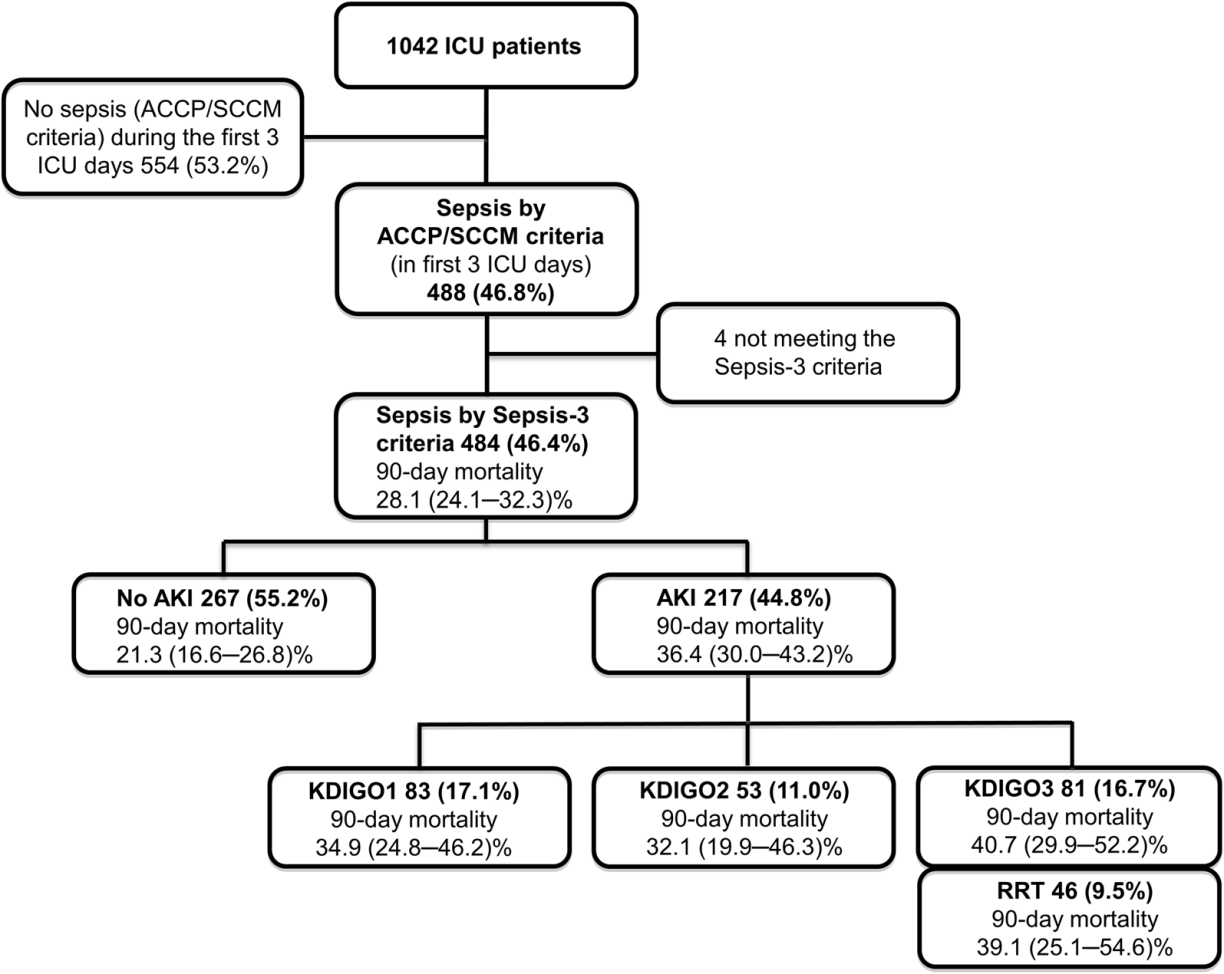
Study flowchart

We included 484 patients fulfilling the Sepsis-3 definition (Fig. 1). Table 1 presents the patient characteristics. Of AKI patients, 115/217 (53%) developed AKI on day 1, 87/217 (40%) on day 2, and 15/217 (7%) on day 3. Of 46 patients treated with RRT during the first 3 days in ICU, 20 (43%) commenced RRT on the first ICU day, 19 (41%) on day 2, and 7 (15%) on day 3. The uNGAL measurement used for prediction of outcomes was the 0-h sample in 460 (95%) of 484 patients, 12-h sample in 9 (2%), and 24-h sample in 15 (3%) patients. The first measured uNGAL was below the detection limit in 48 patients (10%) and above it in 110 patients (23%).

**Table 1:** Characteristics of 484 critically ill sepsis patients

	Data available (of 484)	N (%) or median [IQR]
Age (years)	484	65 [54–75]
Gender (male)	484	310 (64)
Baseline serum/plasma creatinine (µmol/l)	338	78 [62–95]
Co-morbidity		
Chronic obstructive pulmonary disease	476	51 (11)
Hypertension	484	255 (53)
Atherosclerosis	484	64 (13)
Diabetes	484	117 (24)
Systolic heart failure	484	64 (13)
Chronic kidney disease	484	35 (7)
Admission type		
Emergency	482	465 (96)
Surgical	484	123 (25)
Diagnostic group (APACHE II)	484	
Respiratory tract, non-operative		109 (23)
Cardiovascular, non-operative		65 (13)
Sepsis		64 (13)
Gastrointestinal tract, operative		63 (13)
Gastrointestinal tract, non-operative		36 (7)
Neurological, non-operative		26 (5)
Metabolic		23 (5)
Cardiovascular, operative		23 (5)
Other (< 4% each)		75 (15)
Other		
Need for vasopressor (in first 3 ICU days)	484	359 (74)
Highest lactate (day of admission, mmol/l)	484	1.9 [1.4–2.9]
Highest creatinine (day of admission, µmol/l)	476	88 [63–140]
Cumulative diuresis (first 24 h, ml)	482	2042 [1300–2953]
SOFA (highest score, points)	484	8 [6–11]
SAPS II score (points)	484	40 [32–53]
Mechanical ventilation	484	342 (71)
Emergency surgery (< 1 week)	482	118 (24)
Length of stay ICU (days)	484	4.0 [2.2–7.6]
Length of stay hospital (days)	483	12 [7–21]

### AUC

The AUCs for uNGAL predicting AKI, severe AKI, and RRT during the 3 first days in ICU, and death by day 90 are shown in Table 2. Adding uNGAL to the clinical risk model yielded statistically significant model improvement for the outcomes AKI, severe AKI, and RRT (*p* < 0.05 for all), but not for 90-day mortality (Table 2).

**Table 2:** Model improvement with urine NGAL added to the clinical risk models for the endpoints

	AKI	Severe AKI (KDIGO 2–3)	RRT	90-day mortality
Goodness of fit^a^ (clinical risk model)	0.406	0.400	0.973	0.365
Goodness of fit^a^ (new model including uNGAL)	0.395	0.338	0.749	0.990
Events (n)	217	134	46	136
Nonevents (n)	267	350	438	348
AUC				
uNGAL alone	0.690 (0.647 to 0.731)	0.728 (0.686 to 0.767)	0.769 (0.729 to 0.806)	0.600 (0.555 to 0.644)
Clinical risk model	0.717 (0.670 to 0.764)	0.759 (0.710 to 0.809)	0.724 (0.643 to 0.805)	0.797 (0.754 to 0.840)
New risk model (uNGAL added)	0.749 (0.704 to 0.794)	0.799 (0.755 to 0.843)	0.824 (0.761 to 0.886)	0.804 (0.762 to 0.846)
Difference, *p* (clinical model vs new model)	0.017	0.011	0.005	0.27
Category-free NRI (%)				
cfNRI_events_	1.38 (− 12 to 14.77)	22.39 (6.11 to 38.67)	47.83 (21.91 to 73.74)	− 4.41 (− 21.06 to 12.23)
cfNRI_nonevents_	48.69 (38.25 to 59.13)	49.14 (39.99 to 58.29)	46.58 (38.54 to 54.62)	34.48 (24.67 to 44.3)
cfNRI	50.07 (33.01 to 67.13)	71.53 (52.88 to 90.19)	94.4 (67.16 to 121.65)	30.07 (10.71 to 49.43)
IDI and summary statistics				
IDI_events_	0.0248 (0.0099 to 0.0398)	0.0398 (0.0185 to 0.061)	0.0615 (0.0361 to 0.0868)	0.0115 (− 0.0003 to 0.0233)
IDI_nonevents_	0.0202 (0.0092 to 0.0312)	0.0152 (0.0049 to 0.0256)	0.0065 (− 0.0007 to 0.0136)	0.0045 (− 0.0008 to 0.0099)
IDI	0.045 (0.0264 to 0.0637)	0.055 (0.0317 to 0.0782)	0.0679 (0.0413 to 0.0945)	0.016 (0.0032 to 0.0289)
IS_old_	0.5263 (0.5006 to 0.5519)	0.408 (0.3711 to 0.4449)	0.1947 (0.1344 to 0.255)	0.4509 (0.4112 to 0.4905)
IS_new_	0.5511 (0.5226 to 0.5796)	0.4476 (0.4083 to 0.487)	0.2557 (0.1917 to 0.3197)	0.4623 (0.4214 to 0.5032)
IP_old_	0.3849 (0.3651 to 0.4048)	0.2267 (0.2111 to 0.2422)	0.0846 (0.0781 to 0.0911)	0.2146 (0.1968 to 0.2325)
IP_new_	0.3648 (0.3425 to 0.3871)	0.2115 (0.1937 to 0.2293)	0.0782 (0.0693 to 0.0871)	0.2102 (0.192 to 0.2283)

95% confidence intervals are shown in parentheses. ^a^A Hosmer–Lemeshow goodness of fit was used to test calibration of the models. “New” refers to the classification model that includes the new biomarker and “old” refers to the classification model that does not

cfNRI = cfNRI_events_ + cfNRI_nonevents_; IS, integrated sensitivity; IP, integrated 1-specificity; IDI = (IS_new_ – IS_old_) – (IP_new_ – IP_old_)

### cfNRI, IDI, and RAP

**Fig. 2:**
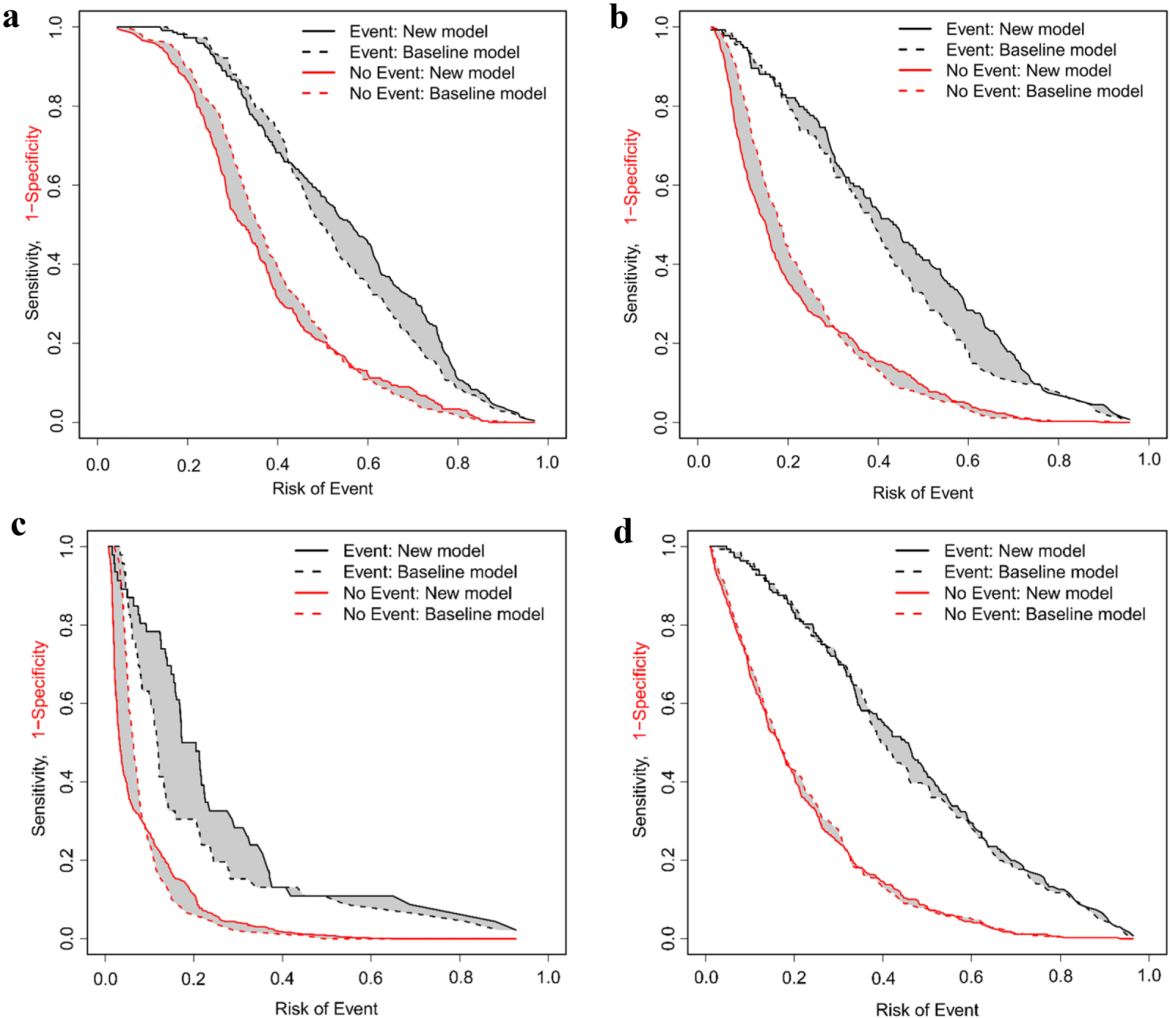
Risk assessment plots showing model enhancement in **a** AKI, **b** severe (KDIGO 2–3) AKI, **c** RRT, and **d** 90-day mortality. Dashed lines (baseline model) represent clinical risk models and solid lines represent new risk models with uNGAL. The gray areas between the solid and the dashed lines represent IDI_events_ (area between black lines) and IDI_nonevents_ (area between red lines). **a** Visually estimated from the curves, adding uNGAL to the clinical risk model improves separation of patients who will develop AKI when the risk of the event is more than ≈ 45%, and discrimination of patients who will not develop AKI when the risk of the event is less than ≈ 50%. **b** With severe AKI, uNGAL added to the clinical risk model improves distinguishing KDIGO 2–3 patients when the risk of the event (= severe AKI) is more than ≈ 25% and helps separating those with KDIGO stage 0–1 when the risk of the event is less than ≈ 30%. **c** Adding uNGAL to the clinical risk model improves the performance for assigning individuals that will end up with RRT when the risk of the event is lower than ≈ 40%, and enhances discrimination of those not ending up with RRT when the risk of the event is lower than ≈ 10%. **d** Corresponding statistics in Table 2, RAPs for the clinical 90-day mortality risk model and for the new model with uNGAL added illustrate that uNGAL offers only minimal enhancement separating those who will die by day 90 when the risk of the event is > 40%

The combined cfNRI and IDI values indicate that the models changed to the right direction when uNGAL was added. The model improved most prominently for RRT (Table 2, Fig. 2c). RAPs showed variable effects with AKI and severe AKI (Fig. 2 a, b), a more sustained effect with RRT (Fig. 2c), but negligible improvement in 90-day mortality prediction (Fig. 2d).

### Decision curve analysis (DCA) and net benefit

**Fig. 3:**
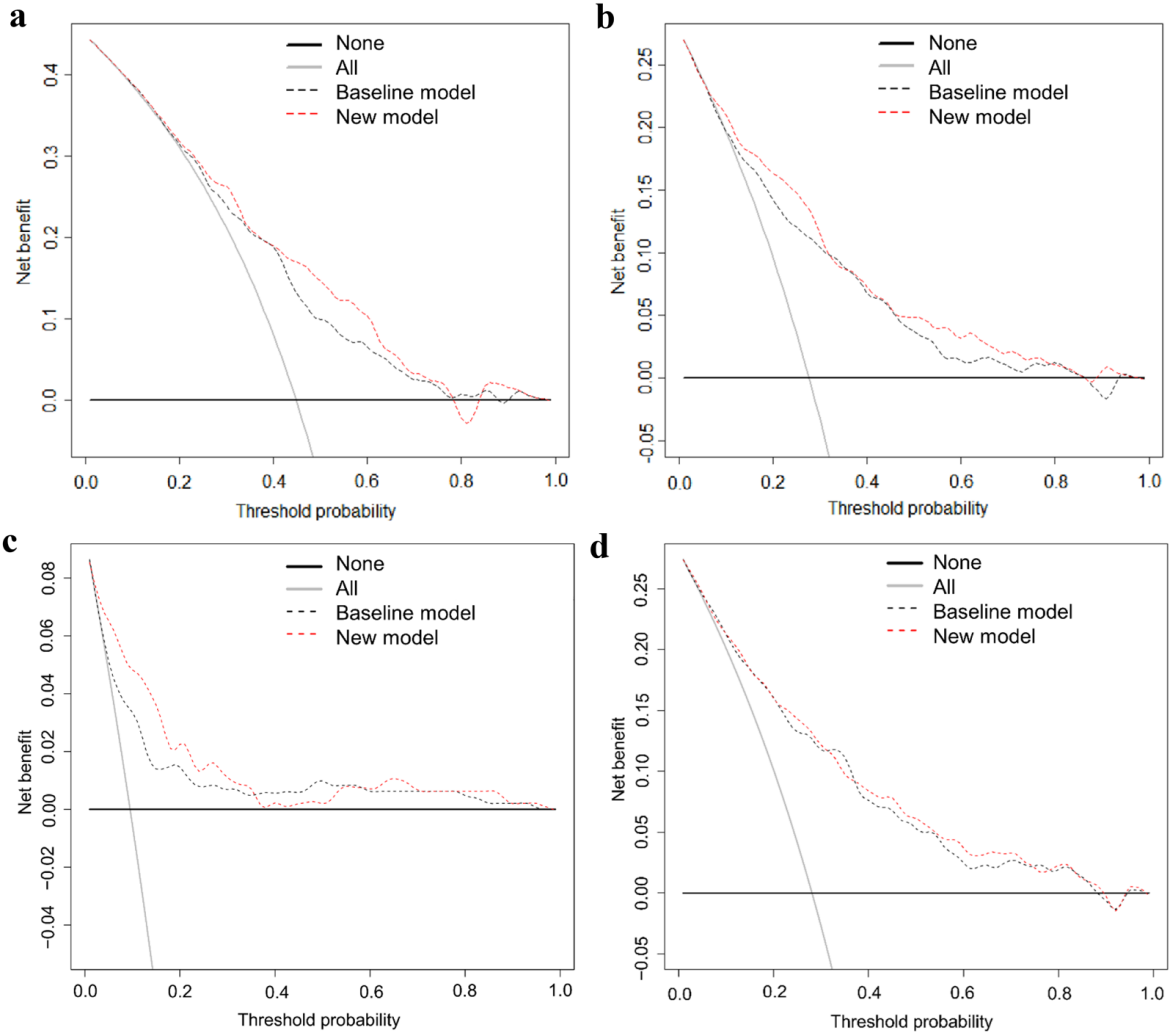
Decision curve analysis for **a** AKI, **b** severe (KDIGO 2–3), AKI, **c** RRT, and **d** 90-day mortality. Dashed black lines (baseline model) represent clinical risk models and dashed red lines represent new models with uNGAL. Black solid line: assume no patient has the outcome. Gray solid line: assume all patients have the outcome. **a** As the new model curve runs higher than the baseline curve, DCA shows a net benefit (NB) in identifying patients who will develop AKI at threshold probabilities of ≈ 0.25–0.35. The magnitude of the NB is 2.5% (95% CI 0.2–4.6%) at the predefined threshold probability of 0.30. However, at a threshold probability of 0.4, there is no NB at all. Note that if the models do not diverge from the gray line of “all expected positive”, neither of them adds anything to the strategy of expecting all to be positive at that threshold probability and should not be used. **b** With severe AKI, there is a 1.4% (95% CI 0.4–4.1%) NB at a threshold probability of 0.2. As with AKI, the NB does not persist within the area of clinically relevant threshold probabilities. **c** Adding uNGAL to the clinical RRT risk model gives a NB of 1.4% (95% CI 0.1–2.8%) in identifying patients who will end up in RRT at a threshold probability of 0.10. Note that at a threshold probability of ≈ 0.35 the curves intersect. **d** Decision curves for the clinical 90-day mortality risk model and for the clinical model including uNGAL do not diverge at a risk threshold of 0.05 thus showing no NB for adding uNGAL to the clinical risk model

At the pre-defined threshold probability of 0.3, there was a 2.5% (95% CI 0.2–4.6%) net benefit of adding uNGAL to the clinical AKI risk model (Fig. 3a). For severe AKI (threshold probability of 0.2) and RRT (threshold probability of 0.1), net benefits were 1.4% (0.4–4.1%, Fig. 3b) and 1.4% (0.1–2.8%, Fig. 3c), respectively. 90-day mortality prediction did not improve at threshold probability of 0.05 (Fig. 3d). The test trade-offs (minimum patient numbers to be tested for one extra true-positive, reciprocal of net benefit) were 40 for AKI, 71 for severe AKI, and 74 for RRT. Repeating the analyses excluding those 24 patients who did not have the 0-h sample did not change the results (see Additional file 5).

## Discussion

In this extended statistical analysis comprising 484 critically ill septic patients, we found that uNGAL alone predicts AKI poorly—not better than a clinical prediction model using data on ICU admission. Adding uNGAL to the clinical prediction model improved the prediction of AKI, severe AKI, and RRT slightly, but the magnitude of the improvement is not clinically meaningful. These findings profoundly contradict the conclusions of the current meta-analysis and do not support the usefulness of uNGAL in critically ill septic patients.

### AUCs for uNGAL

In this study of sepsis patients, the first available uNGAL obtained in the ICU presented an AUC of 0.690 for AKI, resembling the AUC of 0.733 for the highest uNGAL of the first 24 h in 1042 non-selected ICU patients from the FINNAKI study [13]. Our result is in disagreement with the pooled AUC (0.90) of the 12 studies included in a recent meta-analysis evaluating performance of NGAL in septic patients [8]. Importantly, the individual studies in the meta-analysis by Zhang et al. [8] were small—the largest one enrolled 168 patients. Generalizability of the meta-analysis may be questioned since nearly two-thirds of a total of 1263 included study patients were from one country, China. Besides, two of the included studies (228 patients) were conducted in the emergency department—not in the ICU. Disease severity and prevalence of comorbidities like chronic kidney disease in the study population may differ from ours. According to the Quality Assessment of Diagnostic Accuracy Studies 2 (QUADAS-2) criteria, the risk of bias was not dealt properly in the majority of the individual studies [8]. Of the included studies, our results were comparable to the Danish study by Hjortrup and colleagues [26], which may result from similarities in case mix and care representing current clinical practice in high-income countries. In the present sepsis cohort, corresponding the original FINNAKI NGAL—substudy [13], the point estimate AUC for uNGAL seemed to be best for prediction of RRT (AUC 0.769). However, according to our results, uNGAL alone is not adequate to predict AKI, RRT, or 90-day mortality in septic patients.

### Improvement of the clinical prediction model

Based on IDI values, adding uNGAL to clinical reference models resulted in improved prediction of the outcomes. IDI and cfNRI values are somewhat difficult to interpret, but RAPs illustrate these metrics in patients with and without the event revealing model improvement or worsening across different risks of the event. The curves showed that model improvement varied depending on the risk of the event.

### Clinical usefulness of uNGAL

The widely used and easily interpreted AUC plots the true-positive rate (sensitivity) against the false-positive rate (1 – specificity) giving us consecutive cut-offs for the predicted risk. To guide decision making in clinical practice, a cut-off for a biomarker (or a decision threshold) is needed to divide patients to low- and high-risk groups, but such a value is not always reported in biomarker studies [8]. AUC enables comparison of the overall performance of different tests for the same condition but does not tell us the ability of a marker to add value to a pre-existing risk prediction model or, if such is lacking, to clinical judgment.

We used DCA graph [10], which illustrates the net benefit of a model in a range of different threshold probabilities of the event. DCA enables the comparison of the clinical and the new model including NGAL. DCA graphs, especially of AKI and severe AKI, show that the increase of net benefit after adding uNGAL to the clinical risk model varies over the range of clinically relevant threshold probabilities. The clinician chooses the optimal threshold probability, balancing between the harm of a false-positive and a false-negative classification. We chose decreasing threshold along with increasing severity of the event. Test trade-offs between 40 (for AKI) and 74 (for RRT), indicating minimum patient numbers to be tested for one extra true-positive, are hardly acceptable as no specific preventive or curative treatment for AKI exists and the criteria for RRT initiation are still under investigation [27].

### Limitations and strengths

Some obvious limitations of the present study need to be considered. First, as this was a post-hoc analysis of a subgroup of septic patients from a larger FINNAKI NGAL study [13], we were not able to influence sample size. Thus our results, especially the predictive value of uNGAL for RRT, must be interpreted with caution due to small number of events. Furthermore, varying clinical practice in the use of RRT in different countries diminishes the generalizability of our findings. However, to the best of our knowledge, this is the largest multicenter cohort of consecutive intensive care patients studying uNGAL in sepsis. Second, although the study patients were originally screened using the former ACCP/SCCM criteria for sepsis, we now included only those with sepsis according to the recent Sepsis-3 definition. This may have led to exclusion of patients fulfilling Sepsis-3 criteria but not the former criteria. Third, some patients may have had existing AKI already at the time of measurement, a problem in all predictive biomarker studies. We performed a sensitivity analysis excluding those 24 patients that did not have a 0-h urine sample but this did not change the results. Fourth, we did not normalize uNGAL for urinary creatinine [28]. Finally, the purpose of the presented clinical risk models was to enable evaluation of the incremental value of uNGAL, that is, what uNGAL adds on clinical reasoning. Importantly, no model should be used in clinical practice before independent external validation. Even though the results of Hosmer–Lemeshow goodness of fit test and restricting the number of variables in the models did not support overfitting, it cannot be ruled out.

Our study has also some strength. To simulate clinical applicability and to perform a fair comparison, we limited the variables for the clinical risk models to those available on ICU admission. We performed an extended statistical analysis to scrutinize clinical usefulness of a suggested AKI biomarker using the most recently proposed statistical methods including variable weighing of false-negatives and false-positives as recommended [25, 29, 30]. We consider our detailed analysis of 484 patients adds significantly on existing combined uNGAL data from 1263 septic patients [8].

## Conclusions

We conclude that in critically ill adult sepsis patients, the performance of uNGAL alone was inadequate in predicting AKI, RRT, and 90-day mortality. The detailed statistical analyses do not support the clinical usefulness of uNGAL in this patient population.

## Supplementary information

**Additional file 1: Table S1.** Associations of variables explored in the univariable models with outcomes.

**Additional file 2:** Correlations between the variables and multicollinearity test results.

**Additional file 3:** Multivariable models and decision curve analysis results.

**Additional file 4:** Statistical methods.

**Additional file 5:** Sensitivity analysis (raw data).

## Data Availability

The datasets used and/or analyzed during the current study are available from the corresponding author on reasonable request.

## Abbreviations

NGAL: Neutrophil gelatinase-associated lipocalin
AKI: Acute kidney injury
uNGAL: Urine neutrophil gelatinase-associated lipocalin
FINNAKI study: Finnish Acute Kidney Injury study
AUC: Area under the receiver operating characteristic curve
KDIGO: Kidney Disease: Improving Global Outcomes
RRT: Renal replacement therapy
RAP: Risk assessment plot
DCA: Decision curve analysis
ACCP/SCCM: American College of Chest Physicians/Society of Critical Care Medicine
Sepsis-3: Third International Consensus Definitions for Sepsis and Septic Shock
ICU: Intensive care unit
ELISA: Enzyme-linked immunosorbent assay
CI: Confidence interval
cfNRI: Category-free net reclassification improvement
IDI: Integrated discrimination improvement
IQR: Interquartile range
QUADAS-2: Quality Assessment of Diagnostic Accuracy Studies 2
APACHE II: Physiologic data including Acute Physiology and Chronic Health Evaluation II
SAPS II: Simplified Acute Physiology Score
SOFA: Sequential Organ Failure Assessment
