# Supplementary material for: Urine NGAL as a biomarker for septic AKI: a critical appraisal of clinical utility—data from the observational FINNAKI study
Source: Ann Intensive Care. 2020 Apr 28;10:51. doi: 10.1186/s13613-020-00667-7 (PMC7188747; doi:10.1186/s13613-020-00667-7)
Supplement: Supplementary file 1 — Additional file 1: Table S1. Associations of variables explored in the univariable models with outcomes. [file 13613_2020_667_MOESM1_ESM.docx]

**Additional file 1**

**Table S1. Associations of variables explored in the univariable models with outcomes.**

|  | **Data available** (of 484) | **Two-sided p-values for outcomes** | | | |
| --- | --- | --- | --- | --- | --- |
|  |  | **AKI**  **(KDIGO 1-3)** | **Severe AKI (KDIGO 2-3)** | **RRT** | **90-day mortality** |
| Age | 484 | 0.022* | 0.450* | 0.160* | <0.001* |
| Gender (male) | 484 | 0.636* | 0.180* | 0.339 | 0.119* |
| Diabetes | 484 | 0.663* | 0.016* | 0.113* | 0.204* |
| CKD | 484 | 0.016* | 0.023* | 0.003* | 0.009* |
| COPD | 476 | 0.221* | 0.241* | 0.801 | 0.680 |
| Liver disease | 478 | 0.596* | 1.000 | 1.000 | <0.001* |
| Systolic heart failure | 484 | 0.958* | 0.404* | 0.269 | 0.025* |
| Hypertension | 484 | 0.605* | 0.669* | 0.590 | 0.236* |
| Atherosclerosis | 484 | 0.195* | 0.715 | 1.000 | 0.294* |
| ACE or ARB | 477 | 0.295* | 0.783 | 1.000 | 0.792 |
| NSAID | 466 | 0.279* | 0.263* | 0.611 | 0.547* |
| Corticosteroids | 481 | 0.506* | 0.859 | 0.856 | 0.001* |
| Pre-ICU hypovolemia | 484 | <0.001* | <0.001* | 0.003* | 0.598 |
| Pre-ICU diuretics | 484 | 0.040* | 0.020* | 0.700 | 0.184* |
| Pre-ICU colloids | 484 | 0.109* | 0.179* | 0.422 | 0.737 |
| Pre-ICU hypotension | 484 | <0.001* | <0.001* | 0.013* | 0.007* |
| Nonoperative admission | 484 | 0.776* | 0.407* | 0.672 | 0.005* |
| Emergency surgery | 482 | 0.769* | 0.336* | 0.200 | 0.024* |
| SAPS II (-age and renal points) | 484 | 0.192* | 0.491* | 0.466 | <0.001* |
| Highest lactate (day 1) | 484 | <0.001* | <0.001* | 0.002* | <0.001* |
| Acute liver failure | 484 | 0.143* | 0.008* | 0.163 | 0.002* |

Variables with smallest p-values*, restricting the number of covariates to 1 per 8 dependent endpoints, were included in multivariate logistic regression analyses with corresponding endpoints. Note that since the number of endpoint events varies across the outcomes, the number of included variables varies in the four models as well.

*CKD, chronic kidney disease; COPD, chronic obstructive pulmonary disease; ACE, angiotensin convertase enzyme-inhibitor (permanent medication); ARB, angiotensin II receptor blocker (permanent medication); NSAID, non-steroid anti-inflammatory drug (permanent medication); ICU, intensive care unit; colloids, starch or gelatin; SAPS II, Simplified Acute Physiology Score*
