# Supplementary material for: Urine NGAL as a biomarker for septic AKI: a critical appraisal of clinical utility—data from the observational FINNAKI study
Source: Ann Intensive Care. 2020 Apr 28;10:51. doi: 10.1186/s13613-020-00667-7 (PMC7188747; doi:10.1186/s13613-020-00667-7)
Supplement: Supplementary file 3 — Additional file 3: Multivariable models and decision curve analysis results. [file 13613_2020_667_MOESM3_ESM.docx]

1. **AKI**

| **Variables in the Equation** | | | | | | | | | |
| --- | --- | --- | --- | --- | --- | --- | --- | --- | --- |
|  | | B | S.E. | Wald | df | Sig. | Exp(B) | 95% C.I.for EXP(B) | |
|  |  |  |  |  |  |  |  | Lower | Upper |
| Step 1^a^ | Ikä | ,014 | ,007 | 4,091 | 1 | ,043 | 1,014 | 1,000 | 1,028 |
|  | GENDER_MALE(1) | -,145 | ,212 | ,463 | 1 | ,496 | ,865 | ,571 | 1,312 |
|  | ANY_DIABETES(1) | ,140 | ,256 | ,297 | 1 | ,586 | 1,150 | ,696 | 1,900 |
|  | KRMUN(1) | ,806 | ,417 | 3,737 | 1 | ,053 | 2,238 | ,989 | 5,066 |
|  | COPD(1) | -,650 | ,344 | 3,558 | 1 | ,059 | ,522 | ,266 | 1,026 |
|  | LIVER_DISEASE(1) | -1,288 | ,585 | 4,844 | 1 | ,028 | ,276 | ,088 | ,869 |
|  | SYST(1) | -,444 | ,327 | 1,841 | 1 | ,175 | ,641 | ,338 | 1,218 |
|  | RR_TAUTI(1) | -,106 | ,255 | ,172 | 1 | ,678 | ,900 | ,546 | 1,482 |
|  | ASO(1) | ,314 | ,316 | ,989 | 1 | ,320 | 1,369 | ,737 | 2,541 |
|  | ACE_ARB(1) | -,441 | ,262 | 2,837 | 1 | ,092 | ,644 | ,385 | 1,075 |
|  | NSAID_KL(1) | ,378 | ,322 | 1,384 | 1 | ,239 | 1,460 | ,777 | 2,743 |
|  | CHORTICOSTEROID_KL(1) | -,054 | ,341 | ,025 | 1 | ,875 | ,948 | ,485 | 1,850 |
|  | PRE_HYPOVOLEMIA(1) | ,548 | ,225 | 5,928 | 1 | ,015 | 1,731 | 1,113 | 2,691 |
|  | PRE_DIUREETTI(1) | ,450 | ,234 | 3,700 | 1 | ,054 | 1,568 | ,992 | 2,479 |
|  | PRE_COLLOID(1) | -,043 | ,258 | ,028 | 1 | ,867 | ,958 | ,578 | 1,587 |
|  | PRE_HYPOTENSIO(1) | ,594 | ,232 | 6,560 | 1 | ,010 | 1,811 | 1,150 | 2,852 |
|  | NON_OPERATIVE(1) | -,473 | ,348 | 1,845 | 1 | ,174 | ,623 | ,315 | 1,233 |
|  | EMERGENCY_SURGERY(1) | -,395 | ,363 | 1,186 | 1 | ,276 | ,673 | ,331 | 1,372 |
|  | SAPS_wo_age_and_renal_080113 | ,007 | ,009 | ,625 | 1 | ,429 | 1,007 | ,990 | 1,024 |
|  | Highest_LACTATE_onICUADM_DAY | ,112 | ,048 | 5,352 | 1 | ,021 | 1,118 | 1,017 | 1,229 |
|  | ACUTE_LIVER(1) | 1,153 | ,724 | 2,533 | 1 | ,112 | 3,166 | ,766 | 13,093 |
|  | Constant | -1,429 | ,578 | 6,107 | 1 | ,013 | ,240 |  |  |
| a. Variable(s) entered on step 1: Ikä, GENDER_MALE, ANY_DIABETES, KRMUN, COPD, LIVER_DISEASE, SYST, RR_TAUTI, ASO, ACE_ARB, NSAID_KL, CHORTICOSTEROID_KL, PRE_HYPOVOLEMIA, PRE_DIUREETTI, PRE_COLLOID, PRE_HYPOTENSIO, NON_OPERATIVE, EMERGENCY_SURGERY, SAPS_wo_age_and_renal_080113, Highest_LACTATE_onICUADM_DAY, ACUTE_LIVER. | | | | | | | | | |

| **Variables not in the Equation** | | | | | |
| --- | --- | --- | --- | --- | --- |
|  | | | Score | df | Sig. |
| Step 0 | Variables | Ikä | 6,148 | 1 | ,013 |
|  |  | GENDER_MALE(1) | ,324 | 1 | ,569 |
|  |  | ANY_DIABETES(1) | ,295 | 1 | ,587 |
|  |  | KRMUN(1) | 6,650 | 1 | ,010 |
|  |  | COPD(1) | 2,098 | 1 | ,147 |
|  |  | LIVER_DISEASE(1) | ,549 | 1 | ,459 |
|  |  | SYST(1) | ,035 | 1 | ,851 |
|  |  | RR_TAUTI(1) | ,371 | 1 | ,542 |
|  |  | ASO(1) | 2,049 | 1 | ,152 |
|  |  | ACE_ARB(1) | 1,275 | 1 | ,259 |
|  |  | NSAID_KL(1) | 1,515 | 1 | ,218 |
|  |  | CHORTICOSTEROID_KL(1) | ,656 | 1 | ,418 |
|  |  | PRE_HYPOVOLEMIA(1) | 21,671 | 1 | ,000 |
|  |  | PRE_DIUREETTI(1) | 4,628 | 1 | ,031 |
|  |  | PRE_COLLOID(1) | 2,893 | 1 | ,089 |
|  |  | PRE_HYPOTENSIO(1) | 25,231 | 1 | ,000 |
|  |  | NON_OPERATIVE(1) | ,151 | 1 | ,697 |
|  |  | EMERGENCY_SURGERY(1) | ,164 | 1 | ,685 |
|  |  | SAPS_wo_age_and_renal_080113 | 1,979 | 1 | ,160 |
|  |  | Highest_LACTATE_onICUADM_DAY | 18,826 | 1 | ,000 |
|  |  | ACUTE_LIVER(1) | 2,983 | 1 | ,084 |
|  |  | ST_NGAL_FIRST_AVAILABLE | 51,294 | 1 | ,000 |
|  | Overall Statistics | | 89,019 | 22 | ,000 |

1. **Severe AKI**

| **Variables in the Equation** | | | | | | | | | |
| --- | --- | --- | --- | --- | --- | --- | --- | --- | --- |
|  | | B | S.E. | Wald | df | Sig. | Exp(B) | 95% C.I.for EXP(B) | |
|  |  |  |  |  |  |  |  | Lower | Upper |
| Step 1^a^ | Ikä | ,015 | ,008 | 3,761 | 1 | ,052 | 1,016 | 1,000 | 1,032 |
|  | GENDER_MALE(1) | -,421 | ,235 | 3,203 | 1 | ,074 | ,656 | ,414 | 1,041 |
|  | ANY_DIABETES(1) | ,654 | ,272 | 5,771 | 1 | ,016 | 1,923 | 1,128 | 3,279 |
|  | KRMUN(1) | ,565 | ,408 | 1,915 | 1 | ,166 | 1,759 | ,790 | 3,917 |
|  | COPD(1) | -,772 | ,416 | 3,446 | 1 | ,063 | ,462 | ,204 | 1,044 |
|  | SYST(1) | -,076 | ,350 | ,047 | 1 | ,829 | ,927 | ,467 | 1,840 |
|  | RR_TAUTI(1) | -,418 | ,254 | 2,692 | 1 | ,101 | ,659 | ,400 | 1,085 |
|  | NSAID_KL(1) | ,444 | ,347 | 1,636 | 1 | ,201 | 1,559 | ,789 | 3,079 |
|  | PRE_HYPOVOLEMIA(1) | ,978 | ,248 | 15,532 | 1 | ,000 | 2,660 | 1,635 | 4,328 |
|  | PRE_DIUREETTI(1) | ,370 | ,257 | 2,073 | 1 | ,150 | 1,448 | ,875 | 2,398 |
|  | PRE_COLLOID(1) | -,101 | ,285 | ,126 | 1 | ,722 | ,904 | ,517 | 1,580 |
|  | PRE_HYPOTENSIO(1) | ,514 | ,251 | 4,200 | 1 | ,040 | 1,672 | 1,023 | 2,735 |
|  | NON_OPERATIVE(1) | ,060 | ,415 | ,021 | 1 | ,885 | 1,062 | ,471 | 2,397 |
|  | EMERGENCY_SURGERY(1) | -,090 | ,426 | ,045 | 1 | ,832 | ,914 | ,396 | 2,107 |
|  | SAPS_wo_age_and_renal_080113 | -,007 | ,010 | ,488 | 1 | ,485 | ,993 | ,974 | 1,012 |
|  | Highest_LACTATE_onICUADM_DAY | ,117 | ,047 | 6,206 | 1 | ,013 | 1,125 | 1,025 | 1,233 |
|  | ACUTE_LIVER(1) | 1,173 | ,616 | 3,629 | 1 | ,057 | 3,231 | ,967 | 10,797 |
|  | Constant | -2,646 | ,682 | 15,067 | 1 | ,000 | ,071 |  |  |
| a. Variable(s) entered on step 1: Ikä, GENDER_MALE, ANY_DIABETES, KRMUN, COPD, SYST, RR_TAUTI, NSAID_KL, PRE_HYPOVOLEMIA, PRE_DIUREETTI, PRE_COLLOID, PRE_HYPOTENSIO, NON_OPERATIVE, EMERGENCY_SURGERY, SAPS_wo_age_and_renal_080113, Highest_LACTATE_onICUADM_DAY, ACUTE_LIVER. | | | | | | | | | |

| **Variables in the Equation** | | | | | | | | | |
| --- | --- | --- | --- | --- | --- | --- | --- | --- | --- |
|  | | B | S.E. | Wald | df | Sig. | Exp(B) | 95% C.I.for EXP(B) | |
|  |  |  |  |  |  |  |  | Lower | Upper |
| Step 1^a^ | Ikä | ,015 | ,008 | 3,064 | 1 | ,080 | 1,015 | ,998 | 1,032 |
|  | GENDER_MALE(1) | -,254 | ,246 | 1,068 | 1 | ,301 | ,775 | ,479 | 1,256 |
|  | ANY_DIABETES(1) | ,578 | ,283 | 4,177 | 1 | ,041 | 1,783 | 1,024 | 3,103 |
|  | KRMUN(1) | ,491 | ,421 | 1,357 | 1 | ,244 | 1,633 | ,716 | 3,728 |
|  | COPD(1) | -,505 | ,419 | 1,456 | 1 | ,228 | ,603 | ,266 | 1,371 |
|  | SYST(1) | ,131 | ,361 | ,131 | 1 | ,717 | 1,140 | ,562 | 2,312 |
|  | RR_TAUTI(1) | -,282 | ,265 | 1,130 | 1 | ,288 | ,755 | ,449 | 1,268 |
|  | NSAID_KL(1) | ,384 | ,357 | 1,155 | 1 | ,282 | 1,468 | ,729 | 2,958 |
|  | PRE_HYPOVOLEMIA(1) | ,787 | ,257 | 9,358 | 1 | ,002 | 2,197 | 1,327 | 3,637 |
|  | PRE_DIUREETTI(1) | ,226 | ,264 | ,733 | 1 | ,392 | 1,253 | ,747 | 2,102 |
|  | PRE_COLLOID(1) | -,040 | ,295 | ,018 | 1 | ,892 | ,961 | ,539 | 1,714 |
|  | PRE_HYPOTENSIO(1) | ,259 | ,263 | ,971 | 1 | ,324 | 1,295 | ,774 | 2,167 |
|  | NON_OPERATIVE(1) | ,133 | ,422 | ,099 | 1 | ,752 | 1,142 | ,499 | 2,614 |
|  | EMERGENCY_SURGERY(1) | -,118 | ,432 | ,075 | 1 | ,784 | ,888 | ,381 | 2,070 |
|  | SAPS_wo_age_and_renal_080113 | -,006 | ,010 | ,401 | 1 | ,527 | ,994 | ,974 | 1,014 |
|  | Highest_LACTATE_onICUADM_DAY | ,099 | ,049 | 4,147 | 1 | ,042 | 1,105 | 1,004 | 1,216 |
|  | ACUTE_LIVER(1) | 1,406 | ,611 | 5,297 | 1 | ,021 | 4,079 | 1,232 | 13,504 |
|  | ST_NGAL_FIRST_AVAILABLE | ,002 | ,000 | 29,236 | 1 | ,000 | 1,002 | 1,001 | 1,002 |
|  | Constant | -3,310 | ,736 | 20,223 | 1 | ,000 | ,037 |  |  |
| a. Variable(s) entered on step 1: Ikä, GENDER_MALE, ANY_DIABETES, KRMUN, COPD, SYST, RR_TAUTI, NSAID_KL, PRE_HYPOVOLEMIA, PRE_DIUREETTI, PRE_COLLOID, PRE_HYPOTENSIO, NON_OPERATIVE, EMERGENCY_SURGERY, SAPS_wo_age_and_renal_080113, Highest_LACTATE_onICUADM_DAY, ACUTE_LIVER, ST_NGAL_FIRST_AVAILABLE. | | | | | | | | | |

1. **RRT**

| **Variables in the Equation** | | | | | | | | | |
| --- | --- | --- | --- | --- | --- | --- | --- | --- | --- |
|  | | B | S.E. | Wald | df | Sig. | Exp(B) | 95% C.I.for EXP(B) | |
|  |  |  |  |  |  |  |  | Lower | Upper |
| Step 1^a^ | Ikä | ,013 | ,011 | 1,339 | 1 | ,247 | 1,013 | ,991 | 1,036 |
|  | KRMUN(1) | 1,029 | ,468 | 4,834 | 1 | ,028 | 2,799 | 1,118 | 7,004 |
|  | PRE_HYPOVOLEMIA(1) | ,518 | ,366 | 2,004 | 1 | ,157 | 1,679 | ,819 | 3,439 |
|  | PRE_HYPOTENSIO(1) | ,197 | ,360 | ,299 | 1 | ,584 | 1,218 | ,601 | 2,467 |
|  | Highest_LACTATE_onICUADM_DAY | ,164 | ,046 | 12,659 | 1 | ,000 | 1,178 | 1,076 | 1,289 |
|  | ANY_DIABETES(1) | ,311 | ,362 | ,738 | 1 | ,390 | 1,364 | ,671 | 2,772 |
|  | Constant | -4,198 | ,791 | 28,149 | 1 | ,000 | ,015 |  |  |
| a. Variable(s) entered on step 1: Ikä, KRMUN, PRE_HYPOVOLEMIA, PRE_HYPOTENSIO, Highest_LACTATE_onICUADM_DAY, ANY_DIABETES. | | | | | | | | | |

| **Variables in the Equation** | | | | | | | | | |
| --- | --- | --- | --- | --- | --- | --- | --- | --- | --- |
|  | | B | S.E. | Wald | df | Sig. | Exp(B) | 95% C.I.for EXP(B) | |
|  |  |  |  |  |  |  |  | Lower | Upper |
| Step 1^a^ | Ikä | ,016 | ,012 | 1,778 | 1 | ,182 | 1,016 | ,993 | 1,040 |
|  | KRMUN(1) | 1,078 | ,493 | 4,773 | 1 | ,029 | 2,938 | 1,117 | 7,724 |
|  | PRE_HYPOVOLEMIA(1) | ,231 | ,377 | ,376 | 1 | ,540 | 1,260 | ,602 | 2,638 |
|  | PRE_HYPOTENSIO(1) | -,202 | ,376 | ,288 | 1 | ,591 | ,817 | ,391 | 1,708 |
|  | Highest_LACTATE_onICUADM_DAY | ,158 | ,050 | 10,056 | 1 | ,002 | 1,172 | 1,062 | 1,292 |
|  | ANY_DIABETES(1) | ,157 | ,385 | ,166 | 1 | ,684 | 1,170 | ,550 | 2,491 |
|  | ST_NGAL_FIRST_AVAILABLE | ,002 | ,000 | 26,571 | 1 | ,000 | 1,002 | 1,001 | 1,003 |
|  | Constant | -5,328 | ,896 | 35,333 | 1 | ,000 | ,005 |  |  |
| 1. Variable(s) entered on step 1: Ikä, KRMUN, PRE_HYPOVOLEMIA, PRE_HYPOTENSIO, Highest_LACTATE_onICUADM_DAY, ANY_DIABETES, ST_NGAL_FIRST_AVAILABLE. 2. **90-D mortality** | | | | | | | | | |

| **Variables in the Equation** | | | | | | | | | |
| --- | --- | --- | --- | --- | --- | --- | --- | --- | --- |
|  | | B | S.E. | Wald | df | Sig. | Exp(B) | 95% C.I.for EXP(B) | |
|  |  |  |  |  |  |  |  | Lower | Upper |
| Step 1^a^ | Ikä | ,047 | ,010 | 23,335 | 1 | ,000 | 1,048 | 1,028 | 1,068 |
|  | KRMUN(1) | ,701 | ,428 | 2,684 | 1 | ,101 | 2,016 | ,871 | 4,663 |
|  | PRE_HYPOTENSIO(1) | ,200 | ,252 | ,633 | 1 | ,426 | 1,222 | ,746 | 2,001 |
|  | Highest_LACTATE_onICUADM_DAY | ,080 | ,042 | 3,614 | 1 | ,057 | 1,084 | ,998 | 1,177 |
|  | ANY_DIABETES(1) | -,755 | ,296 | 6,492 | 1 | ,011 | ,470 | ,263 | ,840 |
|  | GENDER_MALE(1) | ,470 | ,256 | 3,375 | 1 | ,066 | 1,600 | ,969 | 2,641 |
|  | LIVER_DISEASE(1) | 2,118 | ,578 | 13,417 | 1 | ,000 | 8,318 | 2,678 | 25,841 |
|  | SYST(1) | ,407 | ,338 | 1,448 | 1 | ,229 | 1,502 | ,774 | 2,914 |
|  | RR_TAUTI(1) | ,040 | ,259 | ,024 | 1 | ,878 | 1,041 | ,626 | 1,729 |
|  | ASO(1) | ,038 | ,346 | ,012 | 1 | ,913 | 1,038 | ,528 | 2,044 |
|  | NSAID_KL(1) | -,521 | ,425 | 1,506 | 1 | ,220 | ,594 | ,258 | 1,365 |
|  | CHORTICOSTEROID_KL(1) | ,835 | ,352 | 5,645 | 1 | ,018 | 2,306 | 1,157 | 4,593 |
|  | PRE_DIUREETTI(1) | -,086 | ,272 | ,100 | 1 | ,752 | ,918 | ,539 | 1,563 |
|  | NON_OPERATIVE(1) | ,839 | ,424 | 3,909 | 1 | ,048 | 2,314 | 1,007 | 5,314 |
|  | EMERGENCY_SURGERY(1) | ,046 | ,413 | ,013 | 1 | ,911 | 1,047 | ,466 | 2,355 |
|  | SAPS_wo_age_and_renal_080113 | ,042 | ,010 | 16,590 | 1 | ,000 | 1,043 | 1,022 | 1,064 |
|  | ACUTE_LIVER(1) | ,519 | ,703 | ,544 | 1 | ,461 | 1,680 | ,423 | 6,670 |
|  | Constant | -6,555 | ,872 | 56,525 | 1 | ,000 | ,001 |  |  |
| a. Variable(s) entered on step 1: Ikä, KRMUN, PRE_HYPOTENSIO, Highest_LACTATE_onICUADM_DAY, ANY_DIABETES, GENDER_MALE, LIVER_DISEASE, SYST, RR_TAUTI, ASO, NSAID_KL, CHORTICOSTEROID_KL, PRE_DIUREETTI, NON_OPERATIVE, EMERGENCY_SURGERY, SAPS_wo_age_and_renal_080113, ACUTE_LIVER. | | | | | | | | | |

| **Variables in the Equation** | | | | | | | | | |
| --- | --- | --- | --- | --- | --- | --- | --- | --- | --- |
|  | | B | S.E. | Wald | df | Sig. | Exp(B) | 95% C.I.for EXP(B) | |
|  |  |  |  |  |  |  |  | Lower | Upper |
| Step 1^a^ | Ikä | ,046 | ,010 | 22,263 | 1 | ,000 | 1,047 | 1,027 | 1,067 |
|  | KRMUN(1) | ,663 | ,434 | 2,334 | 1 | ,127 | 1,941 | ,829 | 4,546 |
|  | PRE_HYPOTENSIO(1) | ,002 | ,263 | ,000 | 1 | ,995 | 1,002 | ,598 | 1,678 |
|  | Highest_LACTATE_onICUADM_DAY | ,062 | ,043 | 2,157 | 1 | ,142 | 1,064 | ,979 | 1,157 |
|  | ANY_DIABETES(1) | -,829 | ,303 | 7,505 | 1 | ,006 | ,437 | ,241 | ,790 |
|  | GENDER_MALE(1) | ,601 | ,264 | 5,196 | 1 | ,023 | 1,824 | 1,088 | 3,058 |
|  | LIVER_DISEASE(1) | 2,109 | ,597 | 12,469 | 1 | ,000 | 8,242 | 2,556 | 26,573 |
|  | SYST(1) | ,517 | ,343 | 2,279 | 1 | ,131 | 1,678 | ,857 | 3,284 |
|  | RR_TAUTI(1) | ,109 | ,264 | ,172 | 1 | ,678 | 1,116 | ,666 | 1,870 |
|  | ASO(1) | ,038 | ,349 | ,012 | 1 | ,913 | 1,039 | ,524 | 2,060 |
|  | NSAID_KL(1) | -,523 | ,421 | 1,539 | 1 | ,215 | ,593 | ,260 | 1,354 |
|  | CHORTICOSTEROID_KL(1) | ,832 | ,356 | 5,471 | 1 | ,019 | 2,298 | 1,144 | 4,614 |
|  | PRE_DIUREETTI(1) | -,131 | ,274 | ,228 | 1 | ,633 | ,877 | ,513 | 1,500 |
|  | NON_OPERATIVE(1) | ,917 | ,435 | 4,444 | 1 | ,035 | 2,502 | 1,067 | 5,869 |
|  | EMERGENCY_SURGERY(1) | ,037 | ,421 | ,008 | 1 | ,930 | 1,038 | ,455 | 2,367 |
|  | SAPS_wo_age_and_renal_080113 | ,044 | ,011 | 17,559 | 1 | ,000 | 1,045 | 1,024 | 1,067 |
|  | ACUTE_LIVER(1) | ,637 | ,692 | ,849 | 1 | ,357 | 1,892 | ,488 | 7,340 |
|  | ST_NGAL_FIRST_AVAILABLE | ,001 | ,000 | 8,678 | 1 | ,003 | 1,001 | 1,000 | 1,002 |
|  | Constant | -7,004 | ,910 | 59,198 | 1 | ,000 | ,001 |  |  |
| a. Variable(s) entered on step 1: Ikä, KRMUN, PRE_HYPOTENSIO, Highest_LACTATE_onICUADM_DAY, ANY_DIABETES, GENDER_MALE, LIVER_DISEASE, SYST, RR_TAUTI, ASO, NSAID_KL, CHORTICOSTEROID_KL, PRE_DIUREETTI, NON_OPERATIVE, EMERGENCY_SURGERY, SAPS_wo_age_and_renal_080113, ACUTE_LIVER, ST_NGAL_FIRST_AVAILABLE. | | | | | | | | | |

DCA results:

At threshold probabilities of 0.3, 0.2, 0.1, and 0.05, the gain of true positives and reduction of false positives were -4/188 and 38/484 (Tp 0.3), 2/484 and 32/484 (Tp 0.2), 8/484 and -13/484 (Tp 0.1), and 0/484 and 9/484 (Tp 0.05) for AKI, severe AKI, RRT, and death, respectively.
