# Supplementary material for: Urine NGAL as a biomarker for septic AKI: a critical appraisal of clinical utility—data from the observational FINNAKI study
Source: Ann Intensive Care. 2020 Apr 28;10:51. doi: 10.1186/s13613-020-00667-7 (PMC7188747; doi:10.1186/s13613-020-00667-7)
Supplement: Supplementary file 4 — Additional file 4: Statistical methods. [file 13613_2020_667_MOESM4_ESM.docx]

**Additional file 4**

**Statistical methods**

*Category free net reclassification improvement (cfNRI), integrated discrimination improvement (IDI), and risk assessment plot (RAP)*

In a risk model, each patient received a calculated risk probability from 0 (0%) to 1 (100%). The probabilities of the new risk model including urine NGAL (uNGAL) were compared with the probabilities of the clinical risk model. Each patient was given a value of either +1 or -1 depending on whether the change in the calculated risk was in the correct direction (higher for those with events and lower for those without) or incorrect direction (lower for those with events and higher for those without), respectively. The cfNRI_events_ is the sum of these values among the patients with the outcome event and cfNRI_nonevents_, correspondingly, the sum of the values among the patients without the outcome event. The cfNRI is the sum of the cfNRI_events_ and cfNRI_nonevents_ [1]. We also calculated IDI, which detects both the direction and the quantity of the change between risk models for patients with and without the event. IDI is defined as

IDI = (IS_new_ ̶ IS_old_) ̶ (IP_new_ ̶ IP_old_),

where IS is the integral of sensitivity over all possible cut-off values and IP is the integral of “1 minus specificity”. “New” refers to the classification model including uNGAL and “old” refers to the clinical classification model [2]. We draw RAPs, plotting the portion of patients above certain calculated risk against the certain risk, to visualize how NRI and IDI vary in patients with and without the event according to the risk of the event [3].

**References**

1. Pencina MJ, D'Agostino RB, Sr., Steyerberg EW. Extensions of net reclassification improvement calculations to measure usefulness of new biomarkers. Stat Med. 2011;30(1):11-21.
2. Pencina MJ, D' Agostino RB, D' Agostino RB, Vasan RS. Evaluating the added predictive ability of a new marker: From area under the ROC curve to reclassification and beyond. Stat Med. 2008;27(2):157-72.
3. Pickering JW, Endre ZH. New metrics for assessing diagnostic potential of candidate biomarkers. Clin J Am Soc Nephrol. 2012;7(8):1355-64.
