# Supplementary material for: Urine NGAL as a biomarker for septic AKI: a critical appraisal of clinical utility—data from the observational FINNAKI study
Source: Ann Intensive Care. 2020 Apr 28;10:51. doi: 10.1186/s13613-020-00667-7 (PMC7188747; doi:10.1186/s13613-020-00667-7)
Supplement: Supplementary file 5 — Additional file 5: Sensitivity analysis (raw data). [file 13613_2020_667_MOESM5_ESM.docx]

RAP for AKI

[1,] "Total (n)" "460"

[2,] "Events (n)" "200"

[3,] "Non-events (n)" "260"

[4,] "cfNRI and summary statistics" "-------------------------"

[5,] "cfNRI events (%)" "-2 (-16.01 to 12.01)"

[6,] "cfNRI non-events (%)" "48.46 (37.8 to 59.13)"

[7,] "cfNRI (dimensionless)" "46.46 (28.89 to 64.04)"

[8,] "NRI and summary statistics" "-------------------------"

[9,] "NRI events (%)" "0 (0 to 0)"

[10,] "NRI non-events (%)" "0.38 (-0.39 to 1.15)"

[11,] "NRI (dimensionless)" "0.38 (-0.39 to 1.15)"

[12,] "Weighted NRI and summary statistics" "-------------------------"

[13,] "wNRI (dimensionless)" "NaN (NaN to NaN)"

[14,] "IDI and summary statistics" "-------------------------"

[15,] "IDI events" "0.0247 (0.0094 to 0.0401)"

[16,] "IDI non-events" "0.019 (0.0078 to 0.0302)"

[17,] "IDI" "0.0438 (0.0249 to 0.0626)"

[18,] "Relative IDI (%)" "32.13 (15.48 to 48.78)"

[19,] "IS (null model)" "0.5118 (0.487 to 0.5365)"

[20,] "IS (alt model)" "0.5365 (0.5079 to 0.5651)"

[21,] "IP (null model)" "0.3755 (0.3561 to 0.3949)"

[22,] "IP (alt model)" "0.3565 (0.3349 to 0.3781)"

[23,] "AUC" "-------------------------"

[24,] "AUC (null model)" "0.7139 (0.6668 to 0.7609)"

[25,] "AUC (alt model)" "0.7479 (0.7032 to 0.7926)"

[26,] "difference (P)" "0.0168"

[27,] "Incidence" "0.4348"

>RAP for Severe AKI

[,1] [,2]

[1,] "Total (n)" "460"

[2,] "Events (n)" "121"

[3,] "Non-events (n)" "339"

[4,] "cfNRI and summary statistics" "-------------------------"

[5,] "cfNRI events (%)" "19.01 (1.24 to 36.77)"

[6,] "cfNRI non-events (%)" "49.85 (40.72 to 58.99)"

[7,] "cfNRI (dimensionless)" "68.86 (49.15 to 88.57)"

[8,] "NRI and summary statistics" "-------------------------"

[9,] "NRI events (%)" "2.48 (-1.81 to 6.77)"

[10,] "NRI non-events (%)" "13.26 (8.92 to 17.6)"

[11,] "NRI (dimensionless)" "15.74 (9.76 to 21.72)"

[12,] "Weighted NRI and summary statistics" "-------------------------"

[13,] "wNRI (dimensionless)" "10.43 (7.05 to 13.8)"

[14,] "IDI and summary statistics" "-------------------------"

[15,] "IDI events" "0.0424 (0.0196 to 0.0651)"

[16,] "IDI non-events" "0.0151 (0.0049 to 0.0254)"

[17,] "IDI" "0.0575 (0.0326 to 0.0824)"

[18,] "Relative IDI (%)" "32.55 (14.71 to 50.4)"

[19,] "IS (null model)" "0.3931 (0.3533 to 0.4329)"

[20,] "IS (alt model)" "0.4355 (0.3925 to 0.4785)"

[21,] "IP (null model)" "0.2166 (0.2017 to 0.2316)"

[22,] "IP (alt model)" "0.2015 (0.1842 to 0.2188)"

[23,] "AUC" "-------------------------"

[24,] "AUC (null model)" "0.7534 (0.7026 to 0.8042)"

[25,] "AUC (alt model)" "0.7974 (0.753 to 0.8419)"

[26,] "difference (P)" "0.0089"

[27,] "Incidence" "0.263"

>RAP for RRT

[,1] [,2]

[1,] "Total (n)" "460"

[2,] "Events (n)" "38"

[3,] "Non-events (n)" "422"

[4,] "cfNRI and summary statistics" "-------------------------"

[5,] "cfNRI events (%)" "42.11 (13.26 to 70.95)"

[6,] "cfNRI non-events (%)" "46.45 (38.2 to 54.69)"

[7,] "cfNRI (dimensionless)" "88.55 (58.35 to 118.75)"

[8,] "NRI and summary statistics" "-------------------------"

[9,] "NRI events (%)" "21.05 (3.99 to 38.12)"

[10,] "NRI non-events (%)" "-8.83 (-13.43 to -4.23)"

[11,] "NRI (dimensionless)" "12.22 (-5.35 to 29.79)"

[12,] "Weighted NRI and summary statistics" "-------------------------"

[13,] "wNRI (dimensionless)" "-6.36 (-10.84 to -1.89)"

[14,] "IDI and summary statistics" "-------------------------"

[15,] "IDI events" "0.0486 (0.0207 to 0.0765)"

[16,] "IDI non-events" "0.0044 (-0.002 to 0.0108)"

[17,] "IDI" "0.053 (0.0243 to 0.0816)"

[18,] "Relative IDI (%)" "44.13 (-13.79 to 102.05)"

[19,] "IS (null model)" "0.1916 (0.1159 to 0.2672)"

[20,] "IS (alt model)" "0.2401 (0.1616 to 0.3186)"

[21,] "IP (null model)" "0.0728 (0.0673 to 0.0783)"

[22,] "IP (alt model)" "0.0685 (0.0605 to 0.0764)"

[23,] "AUC" "-------------------------"

[24,] "AUC (null model)" "0.6946 (0.5897 to 0.7996)"

[25,] "AUC (alt model)" "0.8136 (0.7433 to 0.8839)"

[26,] "difference (P)" "0.0111"

[27,] "Incidence" "0.0826"

RAP for Death

[,1] [,2]

[1,] "Total (n)" "460"

[2,] "Events (n)" "127"

[3,] "Non-events (n)" "333"

[4,] "cfNRI and summary statistics" "-------------------------"

[5,] "cfNRI events (%)" "-5.51 (-23.37 to 12.35)"

[6,] "cfNRI non-events (%)" "39.94 (30.05 to 49.83)"

[7,] "cfNRI (dimensionless)" "34.43 (14.06 to 54.8)"

[8,] "NRI and summary statistics" "-------------------------"

[9,] "NRI events (%)" "0 (-3.05 to 3.05)"

[10,] "NRI non-events (%)" "2.7 (-0.82 to 6.22)"

[11,] "NRI (dimensionless)" "2.7 (-1.9 to 7.3)"

[12,] "Weighted NRI and summary statistics" "-------------------------"

[13,] "wNRI (dimensionless)" "1.95 (-0.7 to 4.61)"

[14,] "IDI and summary statistics" "-------------------------"

[15,] "IDI events" "0.0137 (4e-04 to 0.0269)"

[16,] "IDI non-events" "0.0052 (-8e-04 to 0.0112)"

[17,] "IDI" "0.0189 (0.0043 to 0.0335)"

[18,] "Relative IDI (%)" "8.17 (1.5 to 14.85)"

[19,] "IS (null model)" "0.4435 (0.401 to 0.4861)"

[20,] "IS (alt model)" "0.457 (0.4128 to 0.5012)"

[21,] "IP (null model)" "0.2123 (0.1947 to 0.2298)"

[22,] "IP (alt model)" "0.2071 (0.1891 to 0.2251)"

[23,] "AUC" "-------------------------"

[24,] "AUC (null model)" "0.7948 (0.7499 to 0.8398)"

[25,] "AUC (alt model)" "0.8025 (0.7586 to 0.8463)"

[26,] "difference (P)" "0.3125"

[27,] "Incidence" "0.2761"

DCA analyses

predictor harm.applied probability

1 PredAKI 0 TRUE

2 PredNGALAKI 0 TRUE

$interventions.avoided.per

[1] 100

$net.benefit

threshold all none PredAKI PredNGALAKI PredAKI_sm PredNGALAKI_sm

1 0.01 0.429073342 0 0.429073342 0.429073342 0.4290846344 0.4291012240

2 0.02 0.423247560 0 0.423247560 0.423247560 0.4232407030 0.4232103812

3 0.03 0.417301658 0 0.417301658 0.417301658 0.4172823475 0.4172674742

4 0.04 0.411231884 0 0.411231884 0.411231884 0.4112115030 0.4112732027

5 0.05 0.405034325 0 0.405034325 0.405263158 0.4050213594 0.4052200253

6 0.06 0.398704903 0 0.398704903 0.399121184 0.3987000483 0.3991618973

7 0.07 0.392239364 0 0.392402992 0.393057504 0.3923926830 0.3930892120

8 0.08 0.385633270 0 0.386011342 0.386956522 0.3861942391 0.3868750299

9 0.09 0.378881988 0 0.380172002 0.380602007 0.3799963827 0.3805334752

10 0.10 0.371980676 0 0.373671498 0.373913043 0.3736683232 0.3741477993

11 0.11 0.364924279 0 0.367073766 0.367879824 0.3671072621 0.3678184692

12 0.12 0.357707510 0 0.360375494 0.361264822 0.3605037680 0.3613102849

13 0.13 0.350324838 0 0.353573213 0.354547726 0.3536819681 0.3542309582

14 0.14 0.342770475 0 0.346663296 0.346258847 0.3461116064 0.3464262863

15 0.15 0.335038363 0 0.337468031 0.338363171 0.3376650000 0.3379783597

16 0.16 0.327122153 0 0.328571429 0.329710145 0.3286182771 0.3301346180

17 0.17 0.319015191 0 0.319617601 0.322341540 0.3196362422 0.3232113212

18 0.18 0.310710498 0 0.311081654 0.318239661 0.3109405435 0.3165099230

19 0.19 0.302200751 0 0.303059581 0.309178744 0.3029938841 0.3099118971

20 0.20 0.293478261 0 0.295652174 0.303260870 0.2965189501 0.3039053002

21 0.21 0.284534948 0 0.291717116 0.298514034 0.2915993454 0.2986065859

22 0.22 0.275362319 0 0.286845039 0.294816054 0.2865959332 0.2930515040

23 0.23 0.265951440 0 0.281253529 0.285573123 0.2799035674 0.2871982804

24 0.24 0.256292906 0 0.270366133 0.280434783 0.2716495522 0.2804723701

25 0.25 0.246376812 0 0.263768116 0.276086957 0.2635440168 0.2735945368

26 0.26 0.236192714 0 0.255699177 0.264806110 0.2565767426 0.2682107753

27 0.27 0.225729601 0 0.251101846 0.264592019 0.2496441051 0.2642742325

28 0.28 0.214975845 0 0.241545894 0.261956522 0.2420224109 0.2607352385

29 0.29 0.203919167 0 0.233649724 0.255633803 0.2334199812 0.2548210085

30 0.30 0.192546584 0 0.226397516 0.244099379 0.2263331345 0.2451490501

31 0.31 0.180844360 0 0.218840580 0.235097669 0.2212922150 0.2338970563

32 0.32 0.168797954 0 0.221355499 0.222250639 0.2174805493 0.2238417125

33 0.33 0.156391953 0 0.210934458 0.215022713 0.2147688247 0.2152370410

34 0.34 0.143610013 0 0.213109354 0.209222661 0.2105311282 0.2072994202

35 0.35 0.130434783 0 0.205016722 0.199331104 0.2048360078 0.2007495580

36 0.36 0.116847826 0 0.197282609 0.193885870 0.1979827432 0.1949232470

37 0.37 0.102829538 0 0.190890269 0.192650104 0.1914980634 0.1899841886

38 0.38 0.088359046 0 0.185694250 0.183941094 0.1868239568 0.1862591216

39 0.39 0.073414113 0 0.182751247 0.182038489 0.1804350780 0.1815199021

40 0.40 0.057971014 0 0.170289855 0.178985507 0.1699927534 0.1767792090

41 0.41 0.042004422 0 0.155158438 0.169123066 0.1556937702 0.1729951075

42 0.42 0.025487256 0 0.139730135 0.170839580 0.1401490928 0.1687732470

43 0.43 0.008390542 0 0.126392067 0.164569031 0.1253830885 0.1634004013

44 0.44 -0.009316770 0 0.112888199 0.156366460 0.1135632651 0.1565336721

45 0.45 -0.027667984 0 0.103952569 0.147430830 0.1066838458 0.1505722117

46 0.46 -0.046698873 0 0.104830918 0.147906602 0.1032434069 0.1462858668

47 0.47 -0.066447908 0 0.100779327 0.142780968 0.0987272821 0.1404931273

48 0.48 -0.086956522 0 0.090969900 0.131270903 0.0905768943 0.1320898560

49 0.49 -0.108269395 0 0.079198636 0.121994885 0.0813958550 0.1236992312

50 0.50 -0.130434783 0 0.076086957 0.119565217 0.0756131161 0.1193134575

51 0.51 -0.153504880 0 0.073558119 0.117480035 0.0745016255 0.1179660388

52 0.52 -0.177536232 0 0.076268116 0.118115942 0.0764266861 0.1154805801

53 0.53 -0.202590194 0 0.076919519 0.112257169 0.0761713806 0.1132848308

54 0.54 -0.228733459 0 0.075803403 0.108790170 0.0712170306 0.1132332127

55 0.55 -0.256038647 0 0.059420290 0.117874396 0.0655994336 0.1124296210

56 0.56 -0.284584980 0 0.061264822 0.110276680 0.0613247970 0.1098592714

57 0.57 -0.314459050 0 0.063599596 0.099848332 0.0588373808 0.1042404904

58 0.58 -0.345755694 0 0.052587992 0.101242236 0.0561446107 0.0977774291

59 0.59 -0.378579003 0 0.052757158 0.090668081 0.0525489952 0.0923590320

60 0.60 -0.413043478 0 0.048913043 0.085869565 0.0501164420 0.0847870800

61 0.61 -0.449275362 0 0.047881828 0.075585284 0.0431208736 0.0760872920

62 0.62 -0.487414188 0 0.032837529 0.066475973 0.0324937419 0.0663391579

63 0.63 -0.527614571 0 0.016392479 0.058108108 0.0240098094 0.0567420069

64 0.64 -0.570048309 0 0.026086957 0.045169082 0.0203232460 0.0493664382

65 0.65 -0.614906832 0 0.020496894 0.046583851 0.0223220128 0.0426386103

66 0.66 -0.662404092 0 0.023657289 0.035677749 0.0239832511 0.0358773347

67 0.67 -0.712779974 0 0.027404480 0.028919631 0.0257326828 0.0298103260

68 0.68 -0.766304348 0 0.025000000 0.024184783 0.0284702472 0.0270471135

69 0.69 -0.823281907 0 0.032047686 0.029312763 0.0277659834 0.0264668748

70 0.70 -0.884057971 0 0.023188406 0.025362319 0.0239784489 0.0237513090

71 0.71 -0.949025487 0 0.016341829 0.016266867 0.0175533139 0.0178180855

72 0.72 -1.018633540 0 0.011490683 0.011490683 0.0108216904 0.0112978366

73 0.73 -1.093397746 0 0.006441224 0.008132045 0.0058491362 0.0098380813

74 0.74 -1.173913043 0 0.003010033 0.009030100 0.0043702811 0.0108182151

75 0.75 -1.260869565 0 0.004347826 0.015217391 0.0063821798 0.0087563562

76 0.76 -1.355072464 0 0.015579710 -0.001449275 0.0104460708 0.0014213511

77 0.77 -1.457466919 0 0.011436673 -0.008979206 0.0163163035 -0.0100383905

78 0.78 -1.569169960 0 0.022529644 -0.019367589 0.0215528905 -0.0170450898

79 0.79 -1.691511387 0 0.027122153 -0.019772257 0.0247647637 -0.0173938047

80 0.80 -1.826086957 0 0.023913043 -0.010869565 0.0239805731 -0.0175280320

81 0.81 -1.974828375 0 0.018421053 -0.016475973 0.0195278968 -0.0148883072

82 0.82 -2.140096618 0 0.014975845 -0.016908213 0.0158849291 -0.0074922184

83 0.83 -2.324808184 0 0.013554987 0.011381074 0.0122517775 0.0013761778

84 0.84 -2.532608696 0 0.009782609 0.009782609 0.0075300390 0.0084158787

85 0.85 -2.768115942 0 0.001449275 0.005797101 0.0054106055 0.0121715901

86 0.86 -3.037267081 0 0.006211180 0.017080745 0.0079110579 0.0173358241

87 0.87 -3.347826087 0 0.017391304 0.028260870 0.0130100311 0.0242936859

88 0.88 -3.710144928 0 0.015217391 0.026086957 0.0161842147 0.0254758531

89 0.89 -4.138339921 0 0.015217391 0.019565217 0.0153443274 0.0208755986

90 0.90 -4.652173913 0 0.013043478 0.015217391 0.0135759898 0.0163861306

91 0.91 -5.280193237 0 0.013043478 0.015217391 0.0125109667 0.0150793615

92 0.92 -6.065217391 0 0.010869565 0.015217391 0.0114020768 0.0150989693

93 0.93 -7.074534161 0 0.010869565 0.015217391 0.0103370537 0.0141670741

94 0.94 -8.420289855 0 0.008695652 0.010869565 0.0094846156 0.0115352047

95 0.95 -10.304347826 0 0.008695652 0.008695652 0.0081729445 0.0081582387

96 0.96 -13.130434783 0 0.006521739 0.004347826 0.0056747121 0.0057299983

97 0.97 -17.840579710 0 0.002173913 0.004347826 0.0034343619 0.0035910867

98 0.98 -27.260869565 0 0.000000000 0.002173913 0.0012069521 0.0017873449

99 0.99 -55.521739130 0 0.000000000 0.000000000 -0.0009375222 0.0003143666

$interventions.avoided

threshold PredAKI PredNGALAKI PredAKI_sm PredNGALAKI_sm

1 0.01 0.0000000 0.0000000 0.01820665 0.02162821

2 0.02 0.0000000 0.0000000 -0.01331125 -0.04625129

3 0.03 0.0000000 0.0000000 -0.02888089 -0.01410299

4 0.04 0.0000000 0.0000000 -0.02575326 0.11518929

5 0.05 0.0000000 0.4347826 -0.01331279 0.34257348

6 0.06 0.0000000 0.6521739 0.01380298 0.70493487

7 0.07 0.2173913 1.0869565 0.20260793 1.11260171

8 0.08 0.4347826 1.5217391 0.61871068 1.43149078

9 0.09 1.3043478 1.7391304 1.10759716 1.68587928

10 0.10 1.5217391 1.7391304 1.52320972 1.94870369

11 0.11 1.7391304 2.3913043 1.77759821 2.31276922

12 0.12 1.9565217 2.6086957 2.02918310 2.62697185

13 0.13 2.1739130 2.8260870 2.22584409 2.62491314

14 0.14 2.3913043 2.1428571 2.06189675 2.26974755

15 0.15 1.3768116 1.8840580 1.51878846 1.70780398

16 0.16 0.7608696 1.3586957 0.80507202 1.57912942

17 0.17 0.2941176 1.6240409 0.30584172 2.01804243

18 0.18 0.1690821 3.4299517 0.11965597 2.63594995

19 0.19 0.3661327 2.9748284 0.34585321 3.31522420

20 0.20 0.8695652 3.9130435 1.19840983 4.15715164

21 0.21 2.7018634 5.2587992 2.61186941 5.25964386

22 0.22 4.0711462 6.8972332 3.96579419 6.28506949

23 0.23 5.1228733 6.5689981 4.69427688 7.11819781

24 0.24 4.4565217 7.6449275 4.89232036 7.66113827

25 0.25 5.2173913 8.9130435 5.14795779 8.18333292

26 0.26 5.5518395 8.1438127 5.78169799 9.11742678

27 0.27 6.8599034 10.5072464 6.46502741 10.37797926

28 0.28 6.8322981 12.0807453 6.96367370 11.73977521

29 0.29 7.2788606 12.6611694 7.24700276 12.48048571

30 0.30 7.8985507 12.0289855 7.87899326 12.29739267

31 0.31 8.4572230 12.0757363 8.99199455 11.82777438

32 0.32 11.1684783 11.3586957 10.33309989 11.69696219

33 0.33 11.0737813 11.9038208 11.84339707 11.93848006

34 0.34 13.4910486 12.7365729 12.98809273 12.36562166

35 0.35 13.8509317 12.7950311 13.82367875 13.06176065

36 0.36 14.2995169 13.6956522 14.44314313 13.87236564

37 0.37 14.9941246 15.2937720 15.08657736 14.83629145

38 0.38 15.8810069 15.5949657 16.04354168 15.97076754

39 0.39 17.1014493 16.9899666 16.73088019 16.90938364

40 0.40 16.8478261 18.1521739 16.81811298 17.82366588

41 0.41 16.2831389 18.2926829 16.37756981 18.84804450

42 0.42 15.7763975 20.0724638 15.84124012 19.77515369

43 0.43 15.6420627 20.7027300 15.51982504 20.55492239

44 0.44 15.5533597 21.0869565 15.64399985 21.12474343

45 0.45 16.0869565 21.4009662 16.40406177 21.77543078

46 0.46 17.7882798 22.8449905 17.57866849 22.63937949

47 0.47 18.8575393 23.5938945 18.62853432 23.34233685

48 0.48 19.2753623 23.6413043 19.25224731 23.74847839

49 0.49 19.5119787 23.9662822 19.75086552 24.14800798

50 0.50 20.6521739 25.0000000 20.60066617 24.96420096

51 0.51 21.8158568 26.0358056 21.89649732 26.07301251

52 0.52 23.4280936 27.2909699 23.42662376 27.05505505

53 0.53 24.7867104 27.9204266 24.71751701 28.01570244

54 0.54 25.9420290 28.7520129 25.56941450 29.11741685

55 0.55 25.8102767 30.5928854 26.32192672 30.14061191

56 0.56 27.1739130 31.0248447 27.16922651 31.00043348

57 0.57 28.5202136 31.2547674 28.15643404 31.59204144

58 0.58 28.8455772 32.3688156 29.11122589 32.11217213

59 0.59 29.9742078 32.6086957 29.95393328 32.72539627

60 0.60 30.7971014 33.2608696 30.86479886 33.19209470

61 0.61 31.7854597 33.5566643 31.49265143 33.58794745

62 0.62 31.8863955 33.9481066 31.88202150 33.94515217

63 0.63 31.9496204 34.3995859 32.39853362 34.32145797

64 0.64 33.5326087 34.6059783 33.19845139 34.83897523

65 0.65 34.2140468 35.6187291 34.30764051 35.40310335

66 0.66 35.3425560 35.9617918 35.36122186 35.97790415

67 0.67 36.4568462 36.5314731 36.36956791 36.57962609

68 0.68 37.2378517 37.1994885 37.39693811 37.32479895

69 0.69 38.4278513 38.3049779 38.23494890 38.17040558

70 0.70 38.8819876 38.9751553 38.92130112 38.90971092

71 0.71 39.4304960 39.4274342 39.48520836 39.49867522

72 0.72 40.0603865 40.0603865 40.03496655 40.05620441

73 0.73 40.6789756 40.7415128 40.66165819 40.79735433

74 0.74 41.3513514 41.5628672 41.39775261 41.61642087

75 0.75 42.1739130 42.5362319 42.23721617 42.32042075

76 0.76 43.2837529 42.7459954 43.12026295 42.84654447

77 0.77 43.8763411 43.2665161 44.02052807 43.24468886

78 0.78 44.8940914 43.7123746 44.86144045 43.77558180

79 0.79 45.6851954 44.4386351 45.62051924 44.49459925

80 0.80 46.2500000 45.3804348 46.25793428 45.21721692

81 0.81 46.7552335 45.9366613 46.78432419 45.97909282

82 0.82 47.3064687 46.6065748 47.32429257 46.80403513

83 0.83 47.8941854 47.8496595 47.86796703 47.63635060

84 0.84 48.4265010 48.4265010 48.38980619 48.40862936

85 0.85 48.8746803 48.9514066 48.94548448 49.06892527

86 0.86 49.5449949 49.7219414 49.56562460 49.71635781

87 0.87 50.2848576 50.4472764 50.21445000 50.38367795

88 0.88 50.8003953 50.9486166 50.81618586 50.94628784

89 0.89 51.3361016 51.3898388 51.34026635 51.41173854

90 0.90 51.8357488 51.8599034 51.84166272 51.87315363

91 0.91 52.3506928 52.3721930 52.34543644 52.36814053

92 0.92 52.8355388 52.8733459 52.84016645 52.87063112

93 0.93 53.3309958 53.3637214 53.32699710 53.35601488

94 0.94 53.8020352 53.8159112 53.80622451 53.82164094

95 0.95 54.2791762 54.2791762 54.27614280 54.27735340

96 0.96 54.7373188 54.7282609 54.73537756 54.73385374

97 0.97 55.1837741 55.1904975 55.18953453 55.18706525

98 0.98 55.6344277 55.6388642 55.63747058 55.63714382

99 0.99 56.0825648 56.0825648 56.07954257 56.08402866

> $predictors

predictor harm.applied probability

1 PredSevereAKI 0 TRUE

2 PredNGALSevereAKI 0 TRUE

$interventions.avoided.per

[1] 100

$net.benefit

threshold all none PredSevereAKI PredNGALSevereAKI PredSevereAKI_sm

1 0.01 0.255599473 0 0.255599473 0.255599473 2.561275e-01

2 0.02 0.248003549 0 0.248003549 0.248003549 2.475387e-01

3 0.03 0.240251009 0 0.240251009 0.238077095 2.390973e-01

4 0.04 0.232336957 0 0.230163043 0.230706522 2.308447e-01

5 0.05 0.224256293 0 0.222540046 0.224027460 2.225853e-01

6 0.06 0.216003700 0 0.215356152 0.217761332 2.155926e-01

7 0.07 0.207573633 0 0.208672277 0.209513791 2.089643e-01

8 0.08 0.198960302 0 0.202268431 0.204536862 2.006044e-01

9 0.09 0.190157668 0 0.190683230 0.202078356 1.906166e-01

10 0.10 0.181159420 0 0.179710145 0.197101449 1.814392e-01

11 0.11 0.171958964 0 0.174401563 0.191914998 1.749330e-01

12 0.12 0.162549407 0 0.171936759 0.189031621 1.699900e-01

13 0.13 0.152923538 0 0.164842579 0.182283858 1.649381e-01

14 0.14 0.143073812 0 0.158645096 0.175682508 1.600221e-01

15 0.15 0.132992327 0 0.157033248 0.171994885 1.568299e-01

16 0.16 0.122670807 0 0.154658385 0.168737060 1.545862e-01

17 0.17 0.112100576 0 0.152121530 0.163750655 1.508162e-01

18 0.18 0.101272534 0 0.144432662 0.158748674 1.453068e-01

19 0.19 0.090177134 0 0.138862050 0.149812131 1.389493e-01

20 0.20 0.078804348 0 0.133152174 0.146739130 1.334243e-01

21 0.21 0.067143643 0 0.126912493 0.142377545 1.271654e-01

22 0.22 0.055183946 0 0.122129320 0.140301003 1.191130e-01

23 0.23 0.042913608 0 0.107086392 0.137238848 1.105702e-01

24 0.24 0.030320366 0 0.105034325 0.136956522 1.033978e-01

25 0.25 0.017391304 0 0.097826087 0.126811594 9.983790e-02

26 0.26 0.004112808 0 0.099059929 0.118331375 9.774790e-02

27 0.27 -0.009529482 0 0.095026802 0.110720667 9.413297e-02

28 0.28 -0.023550725 0 0.089130435 0.104106280 8.882060e-02

29 0.29 -0.037966932 0 0.082333129 0.100306185 8.394484e-02

30 0.30 -0.052795031 0 0.081055901 0.100621118 8.194705e-02

31 0.31 -0.068052930 0 0.082829238 0.093698803 8.079617e-02

32 0.32 -0.083759591 0 0.079411765 0.088107417 7.845806e-02

33 0.33 -0.099935107 0 0.073880597 0.082608696 7.628384e-02

34 0.34 -0.116600791 0 0.075164690 0.079380764 7.645628e-02

35 0.35 -0.133779264 0 0.079264214 0.085451505 7.686175e-02

36 0.36 -0.151494565 0 0.074864130 0.077173913 7.357518e-02

37 0.37 -0.169772257 0 0.063906142 0.074534161 6.505557e-02

38 0.38 -0.188639551 0 0.056100982 0.068513324 5.535520e-02

39 0.39 -0.208125445 0 0.046151105 0.064682823 4.885305e-02

40 0.40 -0.228260870 0 0.047101449 0.059420290 4.534873e-02

41 0.41 -0.249078850 0 0.043920413 0.060943257 4.388933e-02

42 0.42 -0.270614693 0 0.042728636 0.064317841 4.330856e-02

43 0.43 -0.292906178 0 0.043173150 0.062128146 4.301891e-02

44 0.44 -0.315993789 0 0.043944099 0.058540373 4.357170e-02

45 0.45 -0.339920949 0 0.043280632 0.055928854 4.407640e-02

46 0.46 -0.364734300 0 0.044363929 0.046376812 4.364452e-02

47 0.47 -0.390484003 0 0.042124692 0.045570139 4.191831e-02

48 0.48 -0.417224080 0 0.038127090 0.040635452 3.857992e-02

49 0.49 -0.445012788 0 0.034228474 0.037681159 3.341445e-02

50 0.50 -0.473913043 0 0.028260870 0.032608696 2.796139e-02

51 0.51 -0.503992902 0 0.022315883 0.034383319 2.426462e-02

52 0.52 -0.535326087 0 0.023369565 0.034239130 2.320791e-02

53 0.53 -0.567992599 0 0.024375578 0.034088807 2.290840e-02

54 0.54 -0.602079395 0 0.022967864 0.030056711 2.241687e-02

55 0.55 -0.637681159 0 0.019323671 0.023671498 2.263109e-02

56 0.56 -0.674901186 0 0.026086957 0.030039526 2.346068e-02

57 0.57 -0.713852376 0 0.024823054 0.030738119 2.437888e-02

58 0.58 -0.754658385 0 0.022981366 0.020703934 2.483730e-02

59 0.59 -0.797454931 0 0.024973489 0.018292683 2.461233e-02

60 0.60 -0.842391304 0 0.026086957 0.018478261 2.425570e-02

61 0.61 -0.889632107 0 0.020903010 0.017892977 2.269068e-02

62 0.62 -0.939359268 0 0.021395881 0.021052632 2.045085e-02

63 0.63 -0.991774383 0 0.018448884 0.030611046 1.934186e-02

64 0.64 -1.047101449 0 0.019323671 0.033333333 1.904957e-02

65 0.65 -1.105590062 0 0.018633540 0.036335404 1.850466e-02

66 0.66 -1.167519182 0 0.017902813 0.033248082 1.733722e-02

67 0.67 -1.233201581 0 0.014953887 0.034519104 1.560988e-02

68 0.68 -1.302989130 0 0.014130435 0.031521739 1.438217e-02

69 0.69 -1.377279102 0 0.013253857 0.028471248 1.313565e-02

70 0.70 -1.456521739 0 0.012318841 0.025362319 1.066074e-02

71 0.71 -1.541229385 0 0.006971514 0.029685157 7.522071e-03

72 0.72 -1.631987578 0 0.005900621 0.024534161 7.576585e-03

73 0.73 -1.729468599 0 0.010628019 0.021497585 1.248731e-02

74 0.74 -1.834448161 0 0.022073579 0.028595318 1.849618e-02

75 0.75 -1.947826087 0 0.021739130 0.028260870 2.095115e-02

76 0.76 -2.070652174 0 0.019202899 0.032608696 2.063539e-02

77 0.77 -2.204158790 0 0.018809074 0.030434783 2.110296e-02

78 0.78 -2.349802372 0 0.026086957 0.030434783 2.321324e-02

79 0.79 -2.509316770 0 0.023913043 0.028260870 2.456696e-02

80 0.80 -2.684782609 0 0.023913043 0.026086957 2.368158e-02

81 0.81 -2.878718535 0 0.021739130 0.023913043 2.239497e-02

82 0.82 -3.094202899 0 0.021739130 0.021739130 2.186736e-02

83 0.83 -3.335038363 0 0.021739130 0.019565217 2.121642e-02

84 0.84 -3.605978261 0 0.019565217 0.017391304 1.957502e-02

85 0.85 -3.913043478 0 0.017391304 0.015217391 1.673057e-02

86 0.86 -4.263975155 0 0.013043478 0.015217391 1.422202e-02

87 0.87 -4.668896321 0 0.013043478 0.015217391 1.303367e-02

88 0.88 -5.141304348 0 0.013043478 0.015217391 1.304838e-02

89 0.89 -5.699604743 0 0.013043478 0.010869565 1.239745e-02

90 0.90 -6.369565217 0 0.010869565 0.008695652 1.047018e-02

91 0.91 -7.188405797 0 0.006521739 0.008695652 7.838312e-03

92 0.92 -8.211956522 0 0.006521739 0.006521739 5.072038e-03

93 0.93 -9.527950311 0 0.002173913 0.006521739 3.357358e-03

94 0.94 -11.282608696 0 0.002173913 0.006521739 2.169011e-03

95 0.95 -13.739130435 0 0.002173913 0.002173913 1.261626e-03

96 0.96 -17.423913043 0 0.000000000 0.002173913 5.727401e-04

97 0.97 -23.565217391 0 0.000000000 0.000000000 1.259997e-04

98 0.98 -35.847826087 0 0.000000000 0.000000000 -8.411776e-05

99 0.99 -72.695652174 0 0.000000000 0.000000000 -2.475876e-05

PredNGALSevereAKI_sm

1 2.558782e-01

2 2.471008e-01

3 2.388510e-01

4 2.310721e-01

5 2.238621e-01

6 2.168700e-01

7 2.103915e-01

8 2.052256e-01

9 2.010027e-01

10 1.971436e-01

11 1.928582e-01

12 1.877929e-01

13 1.822161e-01

14 1.766777e-01

15 1.720179e-01

16 1.683563e-01

17 1.637225e-01

18 1.576225e-01

19 1.514080e-01

20 1.462205e-01

21 1.425564e-01

22 1.404116e-01

23 1.383386e-01

24 1.343198e-01

25 1.275399e-01

26 1.187932e-01

27 1.103937e-01

28 1.048455e-01

29 1.014292e-01

30 9.837599e-02

31 9.407968e-02

32 8.778754e-02

33 8.334740e-02

34 8.201820e-02

35 8.115231e-02

36 7.893757e-02

37 7.417259e-02

38 6.860195e-02

39 6.387904e-02

40 6.122515e-02

41 6.148061e-02

42 6.249434e-02

43 6.237311e-02

44 5.909812e-02

45 5.399065e-02

46 4.898303e-02

47 4.445506e-02

48 4.057443e-02

49 3.698794e-02

50 3.438582e-02

51 3.385692e-02

52 3.432780e-02

53 3.266087e-02

54 2.928240e-02

55 2.809127e-02

56 2.824177e-02

57 2.713243e-02

58 2.341494e-02

59 1.890964e-02

60 1.707749e-02

61 1.869884e-02

62 2.280945e-02

63 2.869663e-02

64 3.355765e-02

65 3.524219e-02

66 3.491696e-02

67 3.359177e-02

68 3.107851e-02

69 2.881591e-02

70 2.761142e-02

71 2.622830e-02

72 2.500214e-02

73 2.454261e-02

74 2.616089e-02

75 2.951524e-02

76 3.118731e-02

77 3.121382e-02

78 3.016362e-02

79 2.826577e-02

80 2.621518e-02

81 2.391304e-02

82 2.173913e-02

83 1.943699e-02

84 1.725818e-02

85 1.574010e-02

86 1.534072e-02

87 1.522720e-02

88 1.391062e-02

89 1.165363e-02

90 9.346586e-03

91 7.778463e-03

92 7.305801e-03

93 6.526641e-03

94 5.210068e-03

95 3.490486e-03

96 1.762980e-03

97 6.500882e-04

98 6.454494e-07

99 -1.458604e-04

$interventions.avoided

threshold PredSevereAKI PredNGALSevereAKI PredSevereAKI_sm PredNGALSevereAKI_sm

1 0.01 0.000000 0.0000000 1.0449478 0.3902515

2 0.02 0.000000 0.0000000 -0.9968103 -2.3965712

3 0.03 0.000000 -7.0289855 -2.3791654 -3.5642521

4 0.04 -5.217391 -3.9130435 -3.0440118 -3.3235565

5 0.05 -3.260870 -0.4347826 -3.3290692 -1.1556288

6 0.06 -1.014493 2.7536232 -1.0613525 1.5889497

7 0.07 1.459627 2.5776398 1.6567180 3.8520960

8 0.08 3.804348 6.4130435 2.0582994 7.0339068

9 0.09 0.531401 12.0531401 0.8193243 10.8449791

10 0.10 -1.304348 14.3478261 0.2945967 14.4161923

11 0.11 1.976285 16.1462451 2.1738097 16.9062071

12 0.12 6.884058 19.4202899 5.3892395 18.5179316

13 0.13 7.976589 19.6488294 8.1346783 19.6811031

14 0.14 9.565217 20.0310559 10.4329944 20.6683758

15 0.15 13.623188 22.1014493 13.4100130 22.0603774

16 0.16 16.793478 24.1847826 16.7036165 23.9376721

17 0.17 19.539642 25.2173913 18.9340306 25.2226282

18 0.18 19.661836 26.1835749 20.1018137 25.7174506

19 0.19 20.755149 25.4233410 20.8089680 26.1307239

20 0.20 21.739130 27.1739130 21.8067764 26.9546365

21 0.21 22.484472 28.3022774 22.5803967 28.3544957

22 0.22 23.735178 30.1778656 22.7057258 30.1909914

23 0.23 21.483932 31.5784499 22.6944906 31.9081530

24 0.24 23.659420 33.7681159 23.1339140 32.9376176

25 0.25 24.130435 32.8260870 24.6887560 33.0834490

26 0.26 27.023411 32.5083612 26.6260131 32.6710823

27 0.27 28.268921 32.5120773 28.0345199 32.4324248

28 0.28 28.975155 32.8260870 28.9369168 33.0059318

29 0.29 29.452774 33.8530735 29.8508486 34.0995852

30 0.30 31.231884 35.7971014 31.4085289 35.2604811

31 0.31 33.583450 36.0028050 33.1196435 36.1031471

32 0.32 34.673913 36.5217391 34.4986039 36.4835323

33 0.33 35.289855 37.0619236 35.7838514 37.2060226

34 0.34 37.225064 38.0434783 37.4381364 38.5292707

35 0.35 39.565217 40.7142857 39.0965566 39.9006093

36 0.36 40.241546 40.6521739 40.0293583 40.9773149

37 0.37 39.788484 41.5981199 40.0177675 41.5482130

38 0.38 39.931350 41.9565217 39.8247630 41.9748667

39 0.39 39.771460 42.6700111 40.1884576 42.5568204

40 0.40 41.304348 43.1521739 41.0311724 43.4186710

41 0.41 42.163309 44.6129374 42.1552224 44.6725471

42 0.42 43.271222 46.2525880 43.3518088 45.9906005

43 0.43 44.550051 47.0626896 44.5251675 47.0962758

44 0.44 45.810277 47.6679842 45.7602407 47.7457421

45 0.45 46.835749 48.3816425 46.9293751 48.1516740

46 0.46 48.024575 48.2608696 47.9378336 48.5709022

47 0.47 48.783534 49.1720629 48.7613782 49.0427557

48 0.48 49.329710 49.6014493 49.3814713 49.5950643

49 0.49 49.880213 50.2395741 49.8017882 50.1737566

50 0.50 50.217391 50.6521739 50.1957516 50.8284281

51 0.51 50.566922 51.7263427 50.7536165 51.6681861

52 0.52 51.571906 52.5752508 51.5469646 52.5758599

53 0.53 52.530763 53.3921247 52.4006091 53.2727360

54 0.54 53.244767 53.8486312 53.2035694 53.7928326

55 0.55 53.754941 54.1106719 54.0221392 54.4658870

56 0.56 55.077640 55.3881988 54.8661881 55.2345897

57 0.57 55.724638 56.1708619 55.6937341 55.9031312

58 0.58 56.311844 56.1469265 56.4472511 56.3555689

59 0.59 57.151805 56.6875461 57.1229117 56.7317945

60 0.60 57.898551 57.3913043 57.7773344 57.2975459

61 0.61 58.214540 58.0220955 58.3330155 58.0735489

62 0.62 58.884993 58.8639551 58.8280673 58.9654220

63 0.63 59.330573 60.0448585 59.3815340 59.9244125

64 0.64 59.986413 60.7744565 59.9693111 60.7868436

65 0.65 60.535117 61.4882943 60.5271061 61.4331074

66 0.66 61.067194 61.8577075 61.0399122 61.9447321

67 0.67 61.476314 62.4399740 61.5097921 62.3944292

68 0.68 61.982097 62.8005115 61.9920319 62.7829449

69 0.69 62.473220 63.1568998 62.4665236 63.1742724

70 0.70 62.950311 63.5093168 62.8834154 63.6018910

71 0.71 63.236375 64.1641151 63.2658963 64.0238189

72 0.72 63.695652 64.4202899 63.7593129 64.4424569

73 0.73 64.359738 64.7617630 64.4183880 64.8747544

74 0.74 65.229142 65.4582844 65.0984085 65.3674775

75 0.75 65.652174 65.8695652 65.6322289 65.9092994

76 0.76 65.995423 66.4187643 66.0456085 66.3745646

77 0.77 66.400339 66.7476002 66.4654616 66.7713061

78 0.78 67.012263 67.1348941 66.9277797 67.1283617

79 0.79 67.339020 67.4545955 67.3577087 67.4549625

80 0.80 67.717391 67.7717391 67.7142878 67.7755706

81 0.81 68.035427 68.0864198 68.0504375 68.0864210

82 0.82 68.398727 68.3987275 68.4005833 68.3987287

83 0.83 68.753274 68.7087480 68.7414842 68.7066618

84 0.84 69.057971 69.0165631 69.0592729 69.0147398

85 0.85 69.360614 69.3222506 69.3509248 69.3314478

86 0.86 69.625885 69.6612740 69.6457505 69.6615438

87 0.87 69.960020 69.9925037 69.9584737 69.9912906

88 0.88 70.286561 70.3162055 70.2844733 70.2995698

89 0.89 70.605765 70.5788959 70.5981122 70.5900988

90 0.90 70.893720 70.8695652 70.8903012 70.8769245

91 0.91 71.158624 71.1801242 71.1724175 71.1705504

92 0.92 71.465028 71.4650284 71.4528460 71.4711163

93 0.93 71.732118 71.7648434 71.7408863 71.7644114

94 0.94 72.030527 72.0582794 72.0300210 72.0502206

95 0.95 72.322654 72.3226545 72.3175541 72.3305986

96 0.96 72.599638 72.6086957 72.6019006 72.6077385

97 0.97 72.882116 72.8821156 72.8819635 72.8836222

98 0.98 73.158829 73.1588287 73.1580601 73.1577389

99 0.99 73.429952 73.4299517 73.4302673 73.4301971

> $predictors

predictor harm.applied probability

1 PredRRT 0 TRUE

2 PredNGALRRT 0 TRUE

$interventions.avoided.per

[1] 100

$net.benefit

threshold all none PredRRT PredNGALRRT PredRRT_sm PredNGALRRT_sm

1 0.01 0.073342117 0 0.073342117 0.0733421168 7.383392e-02 0.0725833637

2 0.02 0.063886424 0 0.063886424 0.0669032831 6.290350e-02 0.0670323932

3 0.03 0.054235769 0 0.054235769 0.0597265800 5.359363e-02 0.0623083017

4 0.04 0.044384058 0 0.043115942 0.0584239130 4.583032e-02 0.0582757374

5 0.05 0.034324943 0 0.038787185 0.0568649886 3.965563e-02 0.0553714605

6 0.06 0.024051804 0 0.038852914 0.0511100833 3.495796e-02 0.0518241723

7 0.07 0.013557737 0 0.027699860 0.0472650771 3.021782e-02 0.0478489382

8 0.08 0.002835539 0 0.025803403 0.0440453686 2.574994e-02 0.0436906739

9 0.09 -0.008122312 0 0.023244147 0.0404682274 2.343473e-02 0.0389509973

10 0.10 -0.019323671 0 0.023671498 0.0321256039 2.272346e-02 0.0344280677

11 0.11 -0.030776746 0 0.020810943 0.0307278945 2.182255e-02 0.0301761042

12 0.12 -0.042490119 0 0.021739130 0.0281620553 2.097101e-02 0.0264906849

13 0.13 -0.054472764 0 0.018965517 0.0213893053 1.958656e-02 0.0232886747

14 0.14 -0.066734075 0 0.018756320 0.0211324570 1.730660e-02 0.0204493919

15 0.15 -0.079283887 0 0.013171355 0.0195652174 1.470662e-02 0.0196181064

16 0.16 -0.092132505 0 0.013146998 0.0192546584 1.251264e-02 0.0200836831

17 0.17 -0.105290728 0 0.011052907 0.0213200629 1.140813e-02 0.0202967106

18 0.18 -0.118769883 0 0.010763521 0.0199893955 1.087940e-02 0.0197109203

19 0.19 -0.132581857 0 0.010520666 0.0178207193 9.762520e-03 0.0181133545

20 0.20 -0.146739130 0 0.008152174 0.0163043478 8.285966e-03 0.0170215654

21 0.21 -0.161254816 0 0.006411668 0.0165657677 6.852458e-03 0.0163642660

22 0.22 -0.176142698 0 0.006354515 0.0169453735 6.324886e-03 0.0153343213

23 0.23 -0.191417278 0 0.007001694 0.0124505929 6.870170e-03 0.0144037469

24 0.24 -0.207093822 0 0.007780320 0.0144164760 7.962300e-03 0.0141909127

25 0.25 -0.223188406 0 0.009420290 0.0152173913 9.310605e-03 0.0148043308

26 0.26 -0.239717979 0 0.010517039 0.0153936545 1.070573e-02 0.0149088262

27 0.27 -0.256700417 0 0.011762954 0.0134603931 1.136304e-02 0.0142202928

28 0.28 -0.274154589 0 0.011473430 0.0129227053 1.116228e-02 0.0130979976

29 0.29 -0.292100429 0 0.009889773 0.0123698714 1.078467e-02 0.0110478242

30 0.30 -0.310559006 0 0.010559006 0.0074534161 1.022491e-02 0.0080458884

31 0.31 -0.329552615 0 0.010333963 0.0046943919 9.593952e-03 0.0049188522

32 0.32 -0.349104859 0 0.007928389 0.0029411765 8.956527e-03 0.0033527482

33 0.33 -0.369240753 0 0.008760545 0.0034393251 8.372133e-03 0.0032569007

34 0.34 -0.389986825 0 0.008563900 0.0040184453 8.429315e-03 0.0036156130

35 0.35 -0.411371237 0 0.008361204 0.0035117057 8.787054e-03 0.0039867996

36 0.36 -0.433423913 0 0.009375000 0.0042119565 9.018848e-03 0.0040457024

37 0.37 -0.456176674 0 0.009213251 0.0037267081 9.341132e-03 0.0031318340

38 0.38 -0.479663394 0 0.009046283 0.0023842917 9.031129e-03 0.0020374122

39 0.39 -0.503920171 0 0.008873842 -0.0002494654 8.210858e-03 0.0021345341

40 0.40 -0.528985507 0 0.006521739 0.0050724638 7.629309e-03 0.0033237828

41 0.41 -0.554900516 0 0.007848195 0.0048268239 7.430026e-03 0.0046975811

42 0.42 -0.581709145 0 0.007721139 0.0045727136 7.484658e-03 0.0049006452

43 0.43 -0.609458429 0 0.007589626 0.0043096873 7.521775e-03 0.0043096976

44 0.44 -0.638198758 0 0.006987578 0.0040372671 7.676715e-03 0.0038098588

45 0.45 -0.667984190 0 0.008695652 0.0037549407 8.171238e-03 0.0037369538

46 0.46 -0.698872786 0 0.008695652 0.0034621578 8.663993e-03 0.0046153255

47 0.47 -0.730926989 0 0.008695652 0.0070139459 8.796401e-03 0.0058879474

48 0.48 -0.764214047 0 0.008695652 0.0068561873 8.695652e-03 0.0068559122

49 0.49 -0.798806479 0 0.008695652 0.0066922421 8.695652e-03 0.0069107101

50 0.50 -0.834782609 0 0.008695652 0.0065217391 8.695652e-03 0.0065217491

51 0.51 -0.872227152 0 0.008695652 0.0063442768 8.695652e-03 0.0063442879

52 0.52 -0.911231884 0 0.008695652 0.0061594203 8.695652e-03 0.0061594325

53 0.53 -0.951896392 0 0.008695652 0.0059666975 8.695652e-03 0.0060949370

54 0.54 -0.994328922 0 0.008695652 0.0057655955 8.695652e-03 0.0054305647

55 0.55 -1.038647343 0 0.008695652 0.0055555556 8.695652e-03 0.0048725410

56 0.56 -1.084980237 0 0.008695652 0.0031620553 8.695652e-03 0.0055412010

57 0.57 -1.133468150 0 0.008695652 0.0086956522 8.695652e-03 0.0070084665

58 0.58 -1.184265010 0 0.008695652 0.0086956522 8.695652e-03 0.0085669518

59 0.59 -1.237539767 0 0.008695652 0.0086956522 8.695652e-03 0.0090220455

60 0.60 -1.293478261 0 0.008695652 0.0086956522 8.695652e-03 0.0086956522

61 0.61 -1.352285396 0 0.008695652 0.0086956522 8.695652e-03 0.0086956522

62 0.62 -1.414187643 0 0.008695652 0.0086956522 8.823878e-03 0.0086956522

63 0.63 -1.479435958 0 0.008695652 0.0086956522 8.700554e-03 0.0086956522

64 0.64 -1.548309179 0 0.008695652 0.0086956522 8.039817e-03 0.0086956522

65 0.65 -1.621118012 0 0.006521739 0.0086956522 7.177575e-03 0.0086956522

66 0.66 -1.698209719 0 0.006521739 0.0086956522 6.516837e-03 0.0086956522

67 0.67 -1.779973650 0 0.006521739 0.0086956522 6.393513e-03 0.0086956522

68 0.68 -1.866847826 0 0.006521739 0.0086956522 6.521739e-03 0.0086956522

69 0.69 -1.959326788 0 0.006521739 0.0086956522 6.521739e-03 0.0086956522

70 0.70 -2.057971014 0 0.006521739 0.0086956522 6.521739e-03 0.0086956522

71 0.71 -2.163418291 0 0.006521739 0.0086956522 6.521739e-03 0.0088238781

72 0.72 -2.276397516 0 0.006521739 0.0086956522 6.521739e-03 0.0087005541

73 0.73 -2.397745572 0 0.006521739 0.0086956522 6.521739e-03 0.0080398166

74 0.74 -2.528428094 0 0.006521739 0.0065217391 6.521739e-03 0.0071775747

75 0.75 -2.669565217 0 0.006521739 0.0065217391 6.521739e-03 0.0065168372

76 0.76 -2.822463768 0 0.006521739 0.0065217391 6.521739e-03 0.0063935132

77 0.77 -2.988657845 0 0.006521739 0.0065217391 6.649965e-03 0.0065217391

78 0.78 -3.169960474 0 0.006521739 0.0065217391 6.526641e-03 0.0065217391

79 0.79 -3.368530021 0 0.006521739 0.0065217391 5.865904e-03 0.0065217391

80 0.80 -3.586956522 0 0.004347826 0.0065217391 5.003662e-03 0.0065217391

81 0.81 -3.828375286 0 0.004347826 0.0065217391 4.342924e-03 0.0065217391

82 0.82 -4.096618357 0 0.004347826 0.0065217391 4.219600e-03 0.0065217391

83 0.83 -4.396419437 0 0.004347826 0.0065217391 4.347826e-03 0.0065217391

84 0.84 -4.733695652 0 0.004347826 0.0065217391 4.476052e-03 0.0066499651

85 0.85 -5.115942029 0 0.004347826 0.0065217391 4.352728e-03 0.0065266411

86 0.86 -5.552795031 0 0.004347826 0.0065217391 3.691991e-03 0.0058659036

87 0.87 -6.056856187 0 0.002173913 0.0043478261 2.829749e-03 0.0050036616

88 0.88 -6.644927536 0 0.002173913 0.0043478261 2.169011e-03 0.0044711501

89 0.89 -7.339920949 0 0.002173913 0.0043478261 2.045687e-03 0.0042245021

90 0.90 -8.173913043 0 0.002173913 0.0043478261 2.173913e-03 0.0036919905

91 0.91 -9.193236715 0 0.002173913 0.0021739130 2.173913e-03 0.0028297486

92 0.92 -10.467391304 0 0.002173913 0.0021739130 2.173913e-03 0.0021690111

93 0.93 -12.105590062 0 0.002173913 0.0021739130 2.302139e-03 0.0020456871

94 0.94 -14.289855072 0 0.002173913 0.0021739130 2.178815e-03 0.0023021390

95 0.95 -17.347826087 0 0.002173913 0.0021739130 1.518078e-03 0.0021788150

96 0.96 -21.934782609 0 0.000000000 0.0021739130 7.662022e-04 0.0014938725

97 0.97 -29.579710145 0 0.000000000 0.0000000000 2.825092e-04 0.0009069492

98 0.98 -44.869565217 0 0.000000000 0.0000000000 -2.782387e-05 0.0002819256

99 0.99 -90.739130435 0 0.000000000 0.0000000000 -1.110352e-04 -0.0003408381

$interventions.avoided

threshold PredRRT PredNGALRRT PredRRT_sm PredNGALRRT_sm

1 0.01 0.000000 0.00000 0.3976539 -0.1105406

2 0.02 0.000000 14.78261 -1.3801118 12.5300473

3 0.03 0.000000 17.75362 -0.4898055 23.4382364

4 0.04 -3.043478 33.69565 2.8661130 32.3589733

5 0.05 8.478261 42.82609 8.9935042 39.9923371

6 0.06 23.188406 42.39130 16.9398425 44.0963392

7 0.07 18.788820 44.78261 22.7052793 45.5485329

8 0.08 26.413043 47.39130 26.3749290 46.9110938

9 0.09 31.714976 49.13043 31.6559602 47.7714692

10 0.10 38.695652 46.30435 37.8367678 48.4149644

11 0.11 41.739130 49.76285 42.5638653 49.2470070

12 0.12 47.101449 51.81159 46.5192220 50.5926418

13 0.13 49.147157 50.76923 49.5681724 52.1095670

14 0.14 52.515528 53.97516 51.6859515 53.5392870

15 0.15 52.391304 56.01449 53.2924155 55.9983742

16 0.16 55.271739 58.47826 54.9403564 58.8789632

17 0.17 56.803069 61.81586 56.9454352 61.2893319

18 0.18 59.009662 63.21256 59.0517585 63.1161081

19 0.19 61.006865 64.11899 60.6875289 64.2808825

20 0.20 61.956522 65.21739 62.0366273 65.4850666

21 0.21 63.074534 66.89441 63.2494225 66.8050626

22 0.22 64.703557 68.45850 64.6844192 67.9072542

23 0.23 66.427221 68.25142 66.3756883 68.9203594

24 0.24 68.043478 70.14493 68.0968347 70.0527407

25 0.25 69.782609 71.52174 69.7431551 71.3795743

26 0.26 71.220736 72.60870 71.2668201 72.4823488

27 0.27 72.584541 73.04348 72.4795647 73.2486134

28 0.28 73.447205 73.81988 73.3771962 73.8581380

29 0.29 73.935532 74.54273 74.1554149 74.2247396

30 0.30 74.927536 74.20290 74.8466913 74.3599808

31 0.31 75.652174 74.39691 75.4916396 74.4546295

32 0.32 75.869565 74.80978 76.0925706 74.8884828

33 0.33 76.745718 75.66535 76.6649701 75.6207516

34 0.34 77.365729 76.48338 77.3372985 76.4021812

35 0.35 77.950311 77.04969 78.0288120 77.1367766

36 0.36 78.719807 77.80193 78.6543060 77.7687518

37 0.37 79.242068 78.30787 79.2618644 78.2148113

38 0.38 79.736842 78.64989 79.7368586 78.6024491

39 0.39 80.206243 78.77926 80.1066584 79.1463557

40 0.40 80.326087 80.10870 80.4933552 79.8356895

41 0.41 80.980912 80.54613 80.9172380 80.5272135

42 0.42 81.397516 80.96273 81.3646528 81.0140627

43 0.43 81.794742 81.35996 81.7885789 81.3599946

44 0.44 82.114625 81.73913 82.2008212 81.7135164

45 0.45 82.705314 82.10145 82.6388896 82.1004968

46 0.46 83.062382 82.44802 83.0587230 82.5792072

47 0.47 83.404255 83.21462 83.4171010 83.0834714

48 0.48 83.731884 83.53261 83.7319048 83.5336093

49 0.49 84.046140 83.83762 84.0461589 83.8632854

50 0.50 84.347826 84.13043 84.3478430 84.1304512

51 0.51 84.637681 84.41176 84.6376965 84.4117796

52 0.52 84.916388 84.68227 84.9164019 84.6822878

53 0.53 85.184578 84.94258 85.1845902 84.9526631

54 0.54 85.442834 85.19324 85.4428457 85.1675862

55 0.55 85.691700 85.43478 85.6917101 85.3818793

56 0.56 85.931677 85.49689 85.9316866 85.6812659

57 0.57 86.163234 86.16323 86.1632430 86.0312430

58 0.58 86.386807 86.38681 86.3868147 86.3773038

59 0.59 86.602800 86.60280 86.6028077 86.6284529

60 0.60 86.811594 86.81159 86.8116010 86.8116010

61 0.61 87.013542 87.01354 87.0135487 87.0135487

62 0.62 87.208976 87.20898 87.2158864 87.2089819

63 0.63 87.398206 87.39821 87.3981760 87.3982110

64 0.64 87.581522 87.58152 87.5459110 87.5815267

65 0.65 87.642140 87.75920 87.6786955 87.7592019

66 0.66 87.819499 87.93149 87.8189099 87.9314930

67 0.67 87.991564 88.09864 87.9843552 88.0986412

68 0.68 88.158568 88.26087 88.1585715 88.2608732

69 0.69 88.320731 88.41840 88.3207344 88.4184029

70 0.70 88.478261 88.57143 88.4782641 88.5714317

71 0.71 88.631353 88.72015 88.6313564 88.7246551

72 0.72 88.780193 88.86473 88.7801960 88.8646782

73 0.73 88.924955 89.00536 88.9249580 88.9820862

74 0.74 89.065805 89.06580 89.0658074 89.0898042

75 0.75 89.202899 89.20290 89.2029008 89.2024756

76 0.76 89.336384 89.33638 89.3363866 89.3316440

77 0.77 89.466403 89.46640 89.4696108 89.4664052

78 0.78 89.593088 89.59309 89.5930146 89.5930900

79 0.79 89.716566 89.71657 89.6999710 89.7165675

80 0.80 89.782609 89.83696 89.7998226 89.8369582

81 0.81 89.903382 89.95437 89.9030448 89.9543762

82 0.82 90.021209 90.06893 90.0178019 90.0689304

83 0.83 90.136197 90.18072 90.1361984 90.1807243

84 0.84 90.248447 90.28986 90.2503646 90.2917724

85 0.85 90.358056 90.39642 90.3579633 90.3963264

86 0.86 90.465116 90.50051 90.4551475 90.4905368

87 0.87 90.537231 90.56972 90.5477226 90.5802064

88 0.88 90.642292 90.67194 90.6420384 90.6729508

89 0.89 90.744993 90.77186 90.7429063 90.7696702

90 0.90 90.845411 90.86957 90.8454116 90.8629242

91 0.91 90.943622 90.94362 90.9436225 90.9507397

92 0.92 91.039698 91.03970 91.0396984 91.0394839

93 0.93 91.133707 91.13371 91.1342424 91.1322834

94 0.94 91.225717 91.22572 91.2256004 91.2261143

95 0.95 91.315789 91.31579 91.3129174 91.3156705

96 0.96 91.394928 91.40399 91.3988323 91.4018643

97 0.97 91.483640 91.48364 91.4847126 91.4873144

98 0.98 91.570541 91.57054 91.5701402 91.5714308

99 0.99 91.655687 91.65569 91.6553596 91.6544021

> $predictors

predictor harm.applied probability

1 PredDeath 0 TRUE

2 PredNGALDeath 0 TRUE

$interventions.avoided.per

[1] 100

$net.benefit

threshold all none PredDeath PredNGALDeath PredDeath_sm PredNGALDeath_sm

1 0.01 0.268774704 0 0.2687747036 0.268796662 0.2686697132 0.268654847

2 0.02 0.261313221 0 0.2614906832 0.261889973 0.2616770973 0.261994196

3 0.03 0.253697893 0 0.2547064097 0.255378754 0.2548104784 0.255572064

4 0.04 0.245923913 0 0.2483695652 0.249094203 0.2480766346 0.249365935

5 0.05 0.237986270 0 0.2419908467 0.243135011 0.2414582875 0.243464184

6 0.06 0.229879741 0 0.2342275671 0.238066605 0.2356719106 0.237835338

7 0.07 0.221598878 0 0.2290790089 0.231042543 0.2289716159 0.230867584

8 0.08 0.213137996 0 0.2241965974 0.223534972 0.2204637727 0.221973004

9 0.09 0.204491161 0 0.2066889632 0.211227903 0.2109389136 0.213438918

10 0.10 0.195652174 0 0.2038647343 0.206038647 0.2020035067 0.206324542

11 0.11 0.186614558 0 0.1947239863 0.202833415 0.1961586724 0.200981618

12 0.12 0.177371542 0 0.1927865613 0.194565217 0.1913597449 0.196622409

13 0.13 0.167916042 0 0.1865567216 0.192203898 0.1868374865 0.190797113

14 0.14 0.158240647 0 0.1816986855 0.185288170 0.1830608895 0.184103076

15 0.15 0.148337596 0 0.1809462916 0.175575448 0.1788709215 0.177691898

16 0.16 0.138198758 0 0.1740165631 0.172981366 0.1749013482 0.173150520

17 0.17 0.127815610 0 0.1700104767 0.170508119 0.1706526848 0.170241274

18 0.18 0.117179215 0 0.1673382821 0.168504772 0.1666201424 0.166055183

19 0.19 0.106280193 0 0.1625067096 0.159044552 0.1628921482 0.161088224

20 0.20 0.095108696 0 0.1576086957 0.156521739 0.1574262143 0.157711810

21 0.21 0.083654375 0 0.1518436984 0.157319758 0.1500280840 0.156505472

22 0.22 0.071906355 0 0.1410813824 0.155574136 0.1429238117 0.154544169

23 0.23 0.059853190 0 0.1372953134 0.150084698 0.1384534265 0.149425291

24 0.24 0.047482838 0 0.1376430206 0.141533181 0.1368740316 0.143048055

25 0.25 0.034782609 0 0.1369565217 0.137681159 0.1359455680 0.137794689

26 0.26 0.021739130 0 0.1323149236 0.134077556 0.1332247258 0.133757802

27 0.27 0.008338297 0 0.1293329363 0.130613460 0.1279815091 0.129767097

28 0.28 -0.005434783 0 0.1225845411 0.124275362 0.1223840420 0.125768403

29 0.29 -0.019595836 0 0.1162278016 0.121953460 0.1191282994 0.121459434

30 0.30 -0.034161491 0 0.1192546584 0.117701863 0.1183671823 0.116491219

31 0.31 -0.049149338 0 0.1197857593 0.109357278 0.1181612223 0.110647373

32 0.32 -0.064578005 0 0.1153452685 0.104475703 0.1164897711 0.104049988

33 0.33 -0.080467229 0 0.1108046723 0.097826087 0.1111881203 0.097580912

34 0.34 -0.096837945 0 0.1068511199 0.091436100 0.1034171967 0.091370246

35 0.35 -0.113712375 0 0.0901337793 0.085284281 0.0945514475 0.085964471

36 0.36 -0.131114130 0 0.0876358696 0.082472826 0.0848801610 0.081968753

37 0.37 -0.149068323 0 0.0760524500 0.079779158 0.0766475162 0.079926062

38 0.38 -0.167601683 0 0.0696353436 0.078541374 0.0703428726 0.079413597

39 0.39 -0.186742694 0 0.0655381326 0.081111903 0.0660361616 0.079843876

40 0.40 -0.206521739 0 0.0652173913 0.079710145 0.0640839564 0.080867331

41 0.41 -0.226971260 0 0.0621591746 0.081540162 0.0625281259 0.080725491

42 0.42 -0.248125937 0 0.0613193403 0.079910045 0.0614331617 0.079233942

43 0.43 -0.270022883 0 0.0599923722 0.075743707 0.0606711004 0.076926760

44 0.44 -0.292701863 0 0.0610248447 0.074534161 0.0592325330 0.074224314

45 0.45 -0.316205534 0 0.0561264822 0.071739130 0.0568643075 0.071446925

46 0.46 -0.340579710 0 0.0537842190 0.068679549 0.0543337238 0.068196650

47 0.47 -0.365873667 0 0.0530352748 0.064150943 0.0528789515 0.065345235

48 0.48 -0.392140468 0 0.0523411371 0.063712375 0.0523079711 0.063718670

49 0.49 -0.419437340 0 0.0520460358 0.063086104 0.0518423044 0.062601362

50 0.50 -0.447826087 0 0.0500000000 0.060869565 0.0507865100 0.060892788

51 0.51 -0.477373558 0 0.0503105590 0.056654836 0.0484201649 0.056252082

52 0.52 -0.508152174 0 0.0440217391 0.052355072 0.0453721630 0.050132563

53 0.53 -0.540240518 0 0.0422756707 0.041443108 0.0425830200 0.046545906

54 0.54 -0.573724008 0 0.0402646503 0.047542533 0.0399401049 0.045876787

55 0.55 -0.608695652 0 0.0386473430 0.048792271 0.0372836331 0.046659765

56 0.56 -0.645256917 0 0.0322134387 0.044861660 0.0343987347 0.045990892

57 0.57 -0.683518706 0 0.0336198180 0.043731041 0.0321129641 0.043876572

58 0.58 -0.723602484 0 0.0302277433 0.042753623 0.0313821462 0.043124178

59 0.59 -0.765641569 0 0.0313361612 0.044114528 0.0309713980 0.043581639

60 0.60 -0.809782609 0 0.0304347826 0.043478261 0.0297883546 0.044233578

61 0.61 -0.856187291 0 0.0275362319 0.045206243 0.0277686410 0.044391568

62 0.62 -0.905034325 0 0.0248283753 0.042791762 0.0245988161 0.043153654

63 0.63 -0.956521739 0 0.0223266745 0.041245593 0.0224506705 0.039992915

64 0.64 -1.010869565 0 0.0205314010 0.035265700 0.0220896668 0.036389024

65 0.65 -1.068322981 0 0.0245341615 0.033229814 0.0229032213 0.034166502

66 0.66 -1.129156010 0 0.0228900256 0.033503836 0.0233646964 0.032729274

67 0.67 -1.193675889 0 0.0233860343 0.032147563 0.0222247027 0.030476737

68 0.68 -1.262228261 0 0.0195652174 0.026358696 0.0206158393 0.027683227

69 0.69 -1.335203366 0 0.0209677419 0.025315568 0.0211759074 0.026959710

70 0.70 -1.413043478 0 0.0224637681 0.028985507 0.0240264126 0.028315745

71 0.71 -1.496251874 0 0.0296851574 0.030884558 0.0265145556 0.028339257

72 0.72 -1.585403727 0 0.0267080745 0.025465839 0.0274829859 0.026494741

73 0.73 -1.681159420 0 0.0258454106 0.022141707 0.0276278736 0.024808531

74 0.74 -1.784280936 0 0.0289297659 0.027090301 0.0276729180 0.024383885

75 0.75 -1.895652174 0 0.0282608696 0.023913043 0.0275967476 0.024693882

76 0.76 -2.016304348 0 0.0253623188 0.022826087 0.0263652363 0.023434721

77 0.77 -2.147448015 0 0.0245746692 0.021644612 0.0245751611 0.019702354

78 0.78 -2.290513834 0 0.0237154150 0.016007905 0.0230601951 0.015955477

79 0.79 -2.447204969 0 0.0206004141 0.010248447 0.0212619301 0.014264267

80 0.80 -2.619565217 0 0.0195652174 0.017391304 0.0189054761 0.014011310

81 0.81 -2.810068650 0 0.0162471396 0.016247140 0.0167439841 0.015512207

82 0.82 -3.021739130 0 0.0149758454 0.012801932 0.0150462147 0.017354887

83 0.83 -3.258312020 0 0.0135549872 0.021994885 0.0136630152 0.017666378

84 0.84 -3.524456522 0 0.0125000000 0.016847826 0.0118886730 0.016705983

85 0.85 -3.826086957 0 0.0094202899 0.011594203 0.0096364067 0.013610582

86 0.86 -4.170807453 0 0.0062111801 0.010559006 0.0068598844 0.010300705

87 0.87 -4.568561873 0 0.0050167224 0.009364548 0.0037046121 0.008713176

88 0.88 -5.032608696 0 -0.0007246377 0.005797101 0.0004704479 0.006725931

89 0.89 -5.581027668 0 -0.0023715415 0.004150198 -0.0030266079 0.002854002

90 0.90 -6.239130435 0 -0.0065217391 -0.002173913 -0.0061049740 -0.002284758

91 0.91 -7.043478261 0 -0.0089371981 -0.008937198 -0.0086553685 -0.007476825

92 0.92 -8.048913043 0 -0.0119565217 -0.011956522 -0.0144544132 -0.014469119

93 0.93 -9.341614907 0 -0.0158385093 -0.015838509 -0.0176384116 -0.017362352

94 0.94 -11.065217391 0 -0.0253623188 -0.023188406 -0.0120116431 -0.011026077

95 0.95 -13.478260870 0 0.0086956522 0.008695652 -0.0024706844 -0.001149209

96 0.96 -17.097826087 0 0.0065217391 0.008695652 0.0022626121 0.003285638

97 0.97 -23.130434783 0 0.0000000000 0.000000000 0.0039532663 0.004659830

98 0.98 -35.195652174 0 0.0000000000 0.000000000 0.0025112023 0.002785277

99 0.99 -71.391304348 0 0.0000000000 0.000000000 -0.0020243863 -0.002284402

$interventions.avoided

threshold PredDeath PredNGALDeath PredDeath_sm PredNGALDeath_sm

1 0.01 0.0000000 0.2173913 -0.4163468 0.08380288

2 0.02 0.8695652 2.8260870 1.6012301 2.92800912

3 0.03 3.2608696 5.4347826 3.4727851 5.56608738

4 0.04 5.8695652 7.6086957 5.2123007 7.97434329

5 0.05 7.6086957 9.7826087 6.8194896 10.23915042

6 0.06 6.8115942 12.8260870 8.7657927 12.26152196

7 0.07 9.9378882 12.5465839 9.6528230 12.44998611

8 0.08 12.7173913 11.9565217 8.7099670 10.53291039

9 0.09 2.2222222 6.8115942 6.8606562 9.13285942

10 0.10 7.3913043 9.3478261 5.6420038 9.46118174

11 0.11 6.5612648 13.1225296 7.5184234 11.50149864

12 0.12 11.3043478 12.6086957 10.2852683 14.09040054

13 0.13 12.4749164 16.2541806 12.6388685 15.32067586

14 0.14 14.4099379 16.6149068 15.2292165 15.96923350

15 0.15 18.4782609 15.4347826 17.2859758 16.70372683

16 0.16 18.8043478 18.2608696 19.3001757 18.25280431

17 0.17 20.6010230 20.8439898 20.9149851 20.64688597

18 0.18 22.8502415 23.3816425 22.4920725 22.33059510

19 0.19 23.9702517 22.4942792 24.1190416 23.42261522

20 0.20 25.0000000 24.5652174 24.9412131 25.00104460

21 0.21 25.6521739 27.7122153 25.0260073 27.32783854

22 0.22 24.5256917 29.6640316 25.2204077 29.30538204

23 0.23 25.9262760 30.2079395 26.2783545 30.03800852

24 0.24 28.5507246 29.7826087 28.2545642 30.28862925

25 0.25 30.6521739 30.8695652 30.3389847 30.88591510

26 0.26 31.4715719 31.9732441 31.7383690 31.86796567

27 0.27 32.7133655 33.0595813 32.3758563 32.83836640

28 0.28 32.9192547 33.3540373 32.8968938 33.73262749

29 0.29 33.2533733 34.6551724 33.9523403 34.52497404

30 0.30 35.7971014 35.4347826 35.5501771 35.15567200

31 0.31 37.6016830 35.2805049 37.2304052 35.57848993

32 0.32 38.2336957 35.9239130 38.4758848 35.83349442

33 0.33 38.8339921 36.1989460 38.9165794 36.15265963

34 0.34 39.5396419 36.5473146 38.8883649 36.54326837

35 0.35 37.8571429 36.9565217 38.6920829 37.08344939

36 0.36 38.8888889 37.9710145 38.4020463 37.88154551

37 0.37 38.3313749 38.9659224 38.4375354 38.98725765

38 0.38 38.7070938 40.1601831 38.8274582 40.29689914

39 0.39 39.4593088 41.8952062 39.5246575 41.69012301

40 0.40 40.7608696 42.9347826 40.5848951 43.10266717

41 0.41 41.6065748 44.3955461 41.6611386 44.27787948

42 0.42 42.7329193 45.3002070 42.7458649 45.21105414

43 0.43 43.7462083 45.8341759 43.8295912 45.99572988

44 0.44 45.0197628 46.7391304 44.7938952 46.69684228

45 0.45 45.5072464 47.4154589 45.6044310 47.38080029

46 0.46 46.2948960 48.0434783 46.3618545 47.99284463

47 0.47 47.2386679 48.4921369 47.2161214 48.62707569

48 0.48 48.1521739 49.3840580 48.1472398 49.37841895

49 0.49 49.0727595 50.2218279 49.0488693 50.16658910

50 0.50 49.7826087 50.8695652 49.8597518 50.86932693

51 0.51 50.6990622 51.3086104 50.5205583 51.27826005

52 0.52 50.9698997 51.7391304 51.0989599 51.54698301

53 0.53 51.6570960 51.5832650 51.6820807 52.03396485

54 0.54 52.3027375 52.9227053 52.2731826 52.76799195

55 0.55 52.9644269 53.7944664 52.8561247 53.61603808

56 0.56 53.2298137 54.2236025 53.4038026 54.32069349

57 0.57 54.0999237 54.8627002 53.9851789 54.87506196

58 0.58 54.5877061 55.4947526 54.6680712 55.51967829

59 0.59 55.3831982 56.2711864 55.3560146 56.23200240

60 0.60 56.0144928 56.8840580 55.9708000 56.93234234

61 0.61 56.5003564 57.6300784 56.5180344 57.57606376

62 0.62 56.9915849 58.0925666 56.9813188 58.11543175

63 0.63 57.4879227 58.5990338 57.4958938 58.53024198

64 0.64 58.0163043 58.8451087 58.1010008 58.91113970

65 0.65 58.8461538 59.3143813 58.7541589 59.36168017

66 0.66 59.3478261 59.8945982 59.3728170 59.85125163

67 0.67 59.9448410 60.3763790 59.8915852 60.29837043

68 0.68 60.3196931 60.6393862 60.3726566 60.70635509

69 0.69 60.9294266 61.1247637 60.9368130 61.19426757

70 0.70 61.5217391 61.8012422 61.5825395 61.76568115

71 0.71 62.3270055 62.3759951 62.1971419 62.27510721

72 0.72 62.6932367 62.6449275 62.7267752 62.69008477

73 0.73 63.1357951 62.9988088 63.2018508 63.09598663

74 0.74 63.7074031 63.6427732 63.6605221 63.54419192

75 0.75 64.1304348 63.9855072 64.1095743 64.01104098

76 0.76 64.4736842 64.3935927 64.5067944 64.41268964

77 0.77 64.8785997 64.7910785 64.8781389 64.73619409

78 0.78 65.2731327 65.0557414 65.2542329 65.05869167

79 0.79 65.5998899 65.3247111 65.6180881 65.42892516

80 0.80 65.9782609 65.9239130 65.9623347 65.83706760

81 0.81 66.2962963 66.2962963 66.3082541 66.27936527

82 0.82 66.6595970 66.6118770 66.6606178 66.70981436

83 0.83 67.0141435 67.1870089 67.0151524 67.09559032

84 0.84 67.3706004 67.4534161 67.3593405 67.45386454

85 0.85 67.6854220 67.7237852 67.6894156 67.76237610

86 0.86 67.9979778 68.0687563 68.0085844 68.06285607

87 0.87 68.3408296 68.4057971 68.3213304 68.39465583

88 0.88 68.6166008 68.7055336 68.6334752 68.71748706

89 0.89 68.9496825 69.0302882 68.9417503 69.01494508

90 0.90 69.2512077 69.2995169 69.2553110 69.30018418

91 0.91 69.5723841 69.5723841 69.5737998 69.58699144

92 0.92 69.8865784 69.8865784 69.8719493 69.87072255

93 0.93 70.1940159 70.1940159 70.1853981 70.18623186

94 0.94 70.4671600 70.4810361 70.5433737 70.54939118

95 0.95 70.9839817 70.9839817 70.9147951 70.92176564

96 0.96 71.2681159 71.2771739 71.2495428 71.25445999

97 0.97 71.5374272 71.5374272 71.5589741 71.56210013

98 0.98 71.8278616 71.8278616 71.8425105 71.84357345

99 0.99 72.1124286 72.1124286 72.1005519 72.09940154

>
